# Pan-cancer analysis reveals Rho kinase addiction as a vulnerability of de-differentiated cancer cells

**DOI:** 10.1016/j.isci.2026.116031

**Published:** 2026-06-02

**Authors:** Jaume Barcelo, Yumiko Teigen, Joshua Alexander James Martin, Samantha George, Dipanwita Das, Joanne Sewell, Anna Perdrix-Rosell, Findlay Bewicke-Copley, Ritobrata Ghose, William Yang, Rachel Brough, Andrew Clear, Wencke Walter, Torsten Haferlach, Ilaria Malanchi, John G. Gribben, Louie N. van de Lagemaat, Kamil R. Kranc, Christopher J. Lord, Ana Rio-Machin, Oscar Maiques, Jude Fitzgibbon, Victoria Sanz-Moreno

**Affiliations:** 1 Cytoskeleton and Cancer Metastasis Laboratory, The Breast Cancer Now Toby Robins Research Centre, The Institute of Cancer Research, London SW3 6JB, UK; 2 Centre for Tumor Microenvironment, Barts Cancer Institute, Queen Mary University of London, London, UK; 3 Tumor-Host Interaction Laboratory, The Francis Crick Institute, London, UK; 4 Centre for Haemato-Oncology, Barts Cancer Institute, Queen Mary University of London, London, UK; 5 Centre for Genomics and Computational Biology, Barts Cancer Institute, Queen Mary University of London, London, UK; 6 Precision Oncology Laboratory, The Breast Cancer Now Toby Robins Research Centre, The Institute of Cancer Research, London SW3 6JB, UK; 7 MLL Munich Leukemia Laboratory, Max-Lebsche-Platz 31, 81377 Munich, Germany; 8 Centre for Genome Enabled Biology and Medicine, University of Aberdeen, Aberdeen, UK; 9 The Institute of Cancer Research, Sutton, London, UK; 10 Experimental Haematology Lab, IIS- Fundación Jimenez Díaz, UAM, Madrid, Spain; 11 Centre for Cancer Biomarkers and Biotherapeutics, Barts Cancer Institute, Queen Mary University of London, London, UK

**Keywords:** therapeutics, cancer, transcriptomics

## Abstract

Although Rho kinase (ROCK) has been studied in tumor progression, the reliance of some cancer cells on ROCK-Myosin II for survival remains poorly understood. Using systematic analysis of ROCK inhibitor sensitivity in hundreds of cancer cell lines, we find that ROCK inhibition reduces survival of highly de-differentiated, invasive cancer cells from solid tumors. Transcriptomic analysis reveals enrichment in epithelial-to-mesenchymal transition, migration, proliferation, and inflammation genes, with reduced expression of differentiation and cell-cell junction genes like E-cadherin (CDH1). Acute myeloid leukemia (AML) shows high ROCK inhibitor response among hematological malignancies. Using *in vitro* and *in vivo* approaches, we validate biomarkers of ROCK inhibitor sensitivity in breast cancer, melanoma, and AML, demonstrating their unique addiction to Rho-ROCK-myosin II signaling for survival. Our work has important pre-clinical implications while cautions against wider use of ROCK inhibitors in patient-derived organoid cultures, where they may deplete important cancer cell populations.

## Highlights

• De-differentiated tumor cells rely on ROCK for survival across cancer types
• E-cadherin loss increases ROCK dependency in epithelial cancer cells
• ROCK-NFκB crosstalk drives survival in non-epithelial cancer cells
• MLL-AF6-rearranged AML cells show strong ROCK dependency

## Graphical abstract

**Figure:**
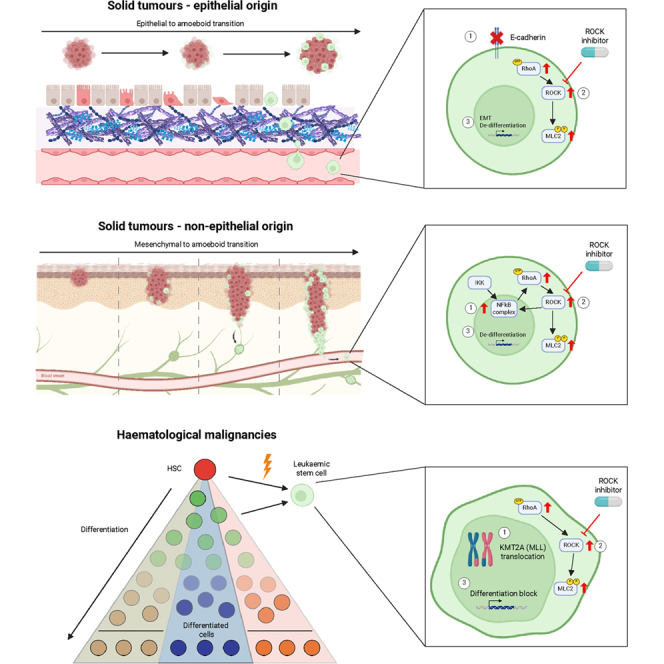

## Introduction

Cancer cells undergo epithelial-to-mesenchymal (EMT) transition, increasing their ability to migrate and invade during metastatic dissemination.^1^ During EMT, cells lose the expression of epithelial markers, such as E-cadherin, and activate mesenchymal markers and transcription factors, such as N-cadherin, vimentin, ZEB1, TWIST1, and SNAIL.^2^ In addition, cancer cells require higher levels of ROCK signaling and actomyosin contractility in order to migrate.^2^ The amoeboid cellular state is part of the EMT spectrum, in which cells can migrate and survive in more challenging environments, and this state is coupled to higher levels of ROCK1/2-Myosin II.^2^

ROCK1 and ROCK2 are involved in multiple cellular processes, including regulation of cell morphology,^3^^,^^4^ cell migration and invasion,^3^^,^^4^^,^^5^^,^^6^ proliferation,^7^ resistance to anoikis,^8^ apoptosis,^9^^,^^10^^,^^11^ and immune response.^12^^,^^13^^,^^14^ Upon activation, ROCK phosphorylates several downstream effectors, including myosin light chain 2 (MLC2)^15^ and myosin phosphatase,^16^ resulting in phosphorylation of MLC2 and increased actomyosin contractility. While the role of ROCK-Myosin II in cancer cell migration and metastasis is widely studied, its function in controlling cancer cell survival is less well-defined. Moreover, whether ROCK controls survival of specific subsets of tumor cells is unclear. Melanoma and breast cancer cells with high levels of Rho-ROCK signaling exhibit cancer stem cell traits.^17^^,^^18^ In these tumor types, the therapy-resistant and highly aggressive features of the disease have been linked to de-differentiation and stemness.^19^^,^^20^^,^^21^^,^^22^ In addition, ROCK inhibition can lead to terminal cell differentiation of chemo-resistant osteosarcoma cancer cells^23^ and sensitize pancreatic stem-like cells to gemcitabine treatment.^24^

ROCK inhibitors are being investigated for the treatment of several diseases, including cancer, glaucoma, asthma, erectile disfunction, insulin resistance, neuronal degeneration, osteoporosis, kidney failure, fibrosis, and graft-versus-host disease (GvHD).^25^^,^^26^ Currently four different ROCK inhibitors have been clinically approved, including three pan-ROCK inhibitors and, more recently, one ROCK2 isoform-specific inhibitor, for the treatment of glaucoma, cerebral vasospasm, hypertension and GvHD.^25^^,^^27^ Nevertheless, the wider approval of ROCK inhibitors in other diseases, such as cancer, has been more challenging, due to side effects and/or poor pharmacodynamic properties of the inhibitors,^25^ as well as the lack of suitable patient stratification biomarkers that could identify those likely to respond well to ROCK inhibition.

The recent development of more selective and potent ROCK inhibitors has the potential to bypass some of these problems.^25^^,^^26^ Specifically in cancer, ROCK inhibitors have been widely studied in diverse preclinical models.^25^^,^^28^^,^^29^ Many studies have reported optimal anti-proliferative and anti-metastatic effects, with studies in melanoma,^7^^,^^17^^,^^30^ acute myeloid leukemia,^31^^,^^32^ pancreatic adenocarcinoma,^33^^,^^34^ breast cancer,^35^^,^^36^^,^^37^ and neuroblastoma.^38^ The use of the dual ROCK/Akt inhibitor AT13148 showed promising effects in preclinical models of melanoma and pancreatic adenocarcinoma,^33^^,^^39^ observations that led to a phase I trial.^40^ However, in this trial, the measurement of a pharmacodynamic biomarker (a change in phosphorylated myosin light chain 2 in pre- vs. post-treatment tumor biopsies) indicated that tumor drug exposure was limited, an observation that was consistent with the lack of anti-tumor effects in this early phase study.^40^

While ongoing efforts are underway to improve the pharmacokinetic properties of ROCK inhibitors, the absence of patient stratification biomarkers (i.e., those that allow the selection of patients who are likely to respond well to a ROCK inhibitor) could also impair the overall utility of this class of drugs. In the present study, we sought to define a set of biomarkers linked to ROCK inhibitor monotherapy response, while simultaneously identifying ROCK-inhibitor responder cellular states.

## Results

### ROCK inhibitor-responder tumor cells are in a unique transcriptional program

To investigate ROCK inhibitor responses at a pan-cancer level, we interrogated data from the publicly available database Genomics of Drug Sensitivity in Cancer (GDSC).^41^ In this study the sensitivity of 978 tumor cell lines to 624 different compounds has been assessed, but to our knowledge the biomarkers that identify tumor cell lines sensitive to ROCK inhibitors included within this dataset have not been identified. We therefore extracted the drug sensitivity data from GDSC for three structurally different ROCK inhibitors: GSK269962, GSK429286, and Y-39983. These are all potent inhibitors of both ROCK1 and ROCK2, but they differ in their off-target profiles, where GSK269962 and GSK429286 are more potent and selective new-generation ROCK inhibitors with lower off-target effects.^25^^,^^26^

**Figure 1:**
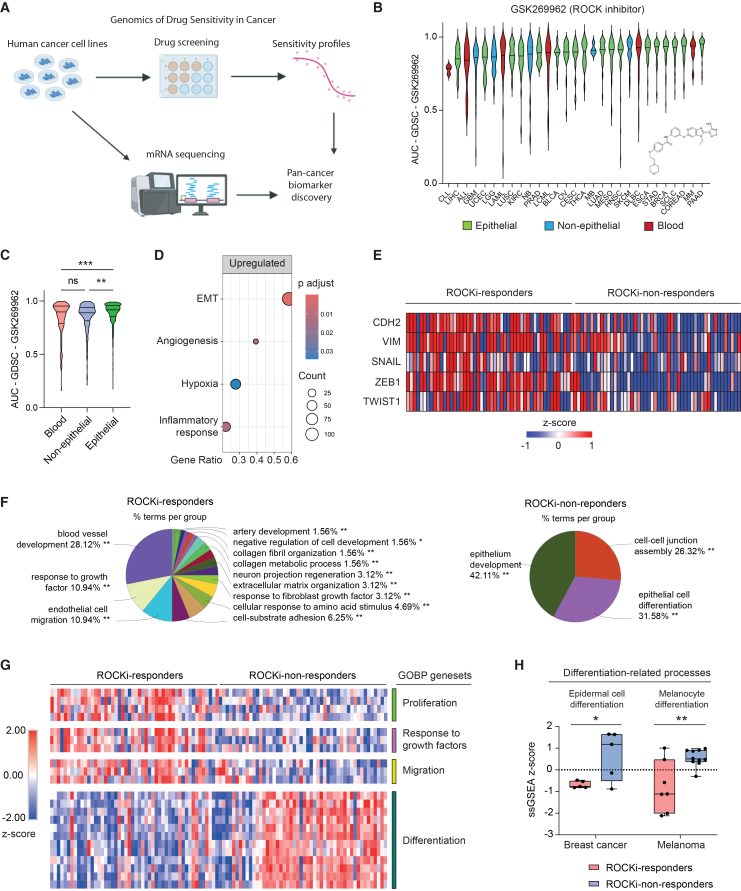
ROCK inhibitor-responder tumor cells are in a unique transcriptional program (A) Schematic of *in silico* analysis. Drug sensitivity data and transcriptomic data were downloaded from GDSC. mRNA expression comparison was performed with the 50 top responder and 50 top non-responder cell lines of solid origin. Schematic created with Biorender.com. (B) Violin plot of pan-cancer GSK269962 sensitivity cell line distribution ranked from lowest to highest AUC response to inhibitor. Red, hematological malignancy; blue, solid tumor of non-epithelial origin; and green, solid tumor of epithelial origin. (C) AUC drug response across all cell lines separated by three categories according to their tumor of origin from (B). Violin plot only, no individual values shown. Statistical test, multiple comparisons by one-way ANOVA. Violin plot shows full distribution with interquartile range. *n* = 143 cell lines (hematological); *n* = 212 cell lines (non-epithelial); and *n* = 550 cell lines (epithelial). (D) Dot plot of GSEA upregulated gene sets from Hallmarks database in GSK269962-responder cells pan-cancer. (E) Heatmap of different EMT markers and EMT-TFs expression in the 100-cell line collection used for differential gene expression analysis. *n* = 50 cell lines (GSK269962 ROCKi-responders) and *n* = 50 cell lines (GSK269962 ROCKi-non-responders). (F) Pie chart of the percentage of upregulated GO terms in ROCK inhibitor-responder or ROCK inhibitor-non responder cell lines from pathway enrichment analysis, visualized in ClueGO.^42^ Gene threshold, DEG >1.3 for ROCK inhibitor-responder or DEG <1.3 for ROCK inhibitor non-responders; *p* value <0.05. (G) ssGSEA of ROCK inhibitor-responder vs. ROCK inhibitor-non-responder cell lines with the GOBP ontology. Values shown in *Z* score per process calculated using the 100-cell line panel. (H) Bar plots of ssGSEA scores in cell lines from breast cancer and melanoma origin separated into responders and non-responders for cell line-specific processes related differentiation from the GOBP ontology. Box shows median value with interquartile range; whiskers show min to max values. Statistical test, multiple *t* test comparisons. For breast cancer, *n* = 5 cell lines (responders) and *n* = 5 cell lines (non-responders). For melanoma, *n* = 7 cell lines (responders) and *n* = 10 cell lines (non-responders). For (C) and (H), ns = not significant, ∗*p* < 0.05, ∗∗*p* < 0.01, ∗∗∗ *p* < 0.001.

We took a pragmatic approach to identify features in the tumor cell line panel associated with sensitivity to ROCK inhibitors and used the area under the curve (AUC) data for each drug and compared this to features such as tumor cell lineage and transcriptomic and genomic profiles across the panel (Figure 1A). When we considered tumor histology, we found that within all cancer types there were very heterogeneous responses to ROCK inhibitors, with responder and non-responder cell lines found in all analyzed tumor types (Figure 1B; Figure S1A). Next, we separated all different cancer types as solid tumors of epithelial origin, solid tumors of non-epithelial origin and hematological malignancies.

Overall, we observed that cell lines from hematological origin were more sensitive to ROCK inhibitors (GSK429286 and Y-39983) when compared to cell lines from solid tumors (Figure S1B). Moreover, within solid tumors, cell lines of non-epithelial origin were overall more responsive to ROCK inhibitors GSK269962 and GSK429286 than those of epithelial origin (Figure 1C; Figure S1B).

We then compared the transcriptomes of GSK269962-responder and non-responder cancer cells from solid tumors (Figure S1C) and observed that responder cancer cells showed an upregulation of genes related to EMT, angiogenesis, hypoxia, and inflammatory response (Figure 1D). Since EMT was the main differentially regulated process in the comparison, we studied the levels of expression of different EMT markers and transcription factors, including *CDH2* (N-cadherin), *VIM* (vimentin), *SNAIL*, *ZEB1*, and *TWIST1*, and observed that they all are significantly upregulated in ROCK inhibitor-responder cancer cell lines (Figures 1E; Figure S1D). Pathway enrichment analysis, using Gene Ontology Biological Processes (GOBP) ontology, revealed that genes related to growth factor responses, blood vessel development and cell migration were highly expressed in ROCK inhibitor-responder cell lines whereas epithelium development, epithelial cell differentiation and cell-cell junction assembly related genes showed higher expression in ROCK inhibitor non-responder cell lines (Figures 1F; Figure S1E). This was further confirmed by single-sample gene set enrichment analysis (ssGSEA) of the 100 pan-cancer cell line collection, where gene sets related to proliferation, response to growth factors, migration, and differentiation were found differentially regulated in responder vs. non-responder cell lines (Figure 1G).

Breast cancer and melanoma were selected for their high metastatic potential for validation in solid tumors of epithelial and non-epithelial origin.^43^^,^^44^ After separating cancer cell lines based on their response to ROCK inhibitor GSK269962, ssGSEA showed that ROCK inhibitor non-responders showed enrichment in terms related to cell differentiation (Figure 1H), in accordance with the pan-cancer analysis in solid tumors.

Overall, our data show that ROCK inhibitor responses are possitively associated with EMT and de-differentiation in solid tumors.

### E-cadherin loss is associated with ROCK inhibitor response in epithelial cancer cells

One key feature of cells undergoing EMT is loss of epithelial markers such as E-cadherin,^45^ while deleterious mutation of the *CDH1* gene, encoding E-cadherin, is a characteristic of tumors such as invasive lobular carcinoma or diffuse gastric carcinoma.^46^^,^^47^ Interestingly, loss of E-cadherin function via somatic inactivation of *CDH1* correlated with increased response to GSK269962 ROCK inhibitor at a pan-cancer level (Figure S2A). Accordingly, lower levels of *CDH1* (E-cadherin) expression were associated with ROCK inhibitor response in the 100 pan-cancer cell line collection (Figure S2B).

**Figure 2:**
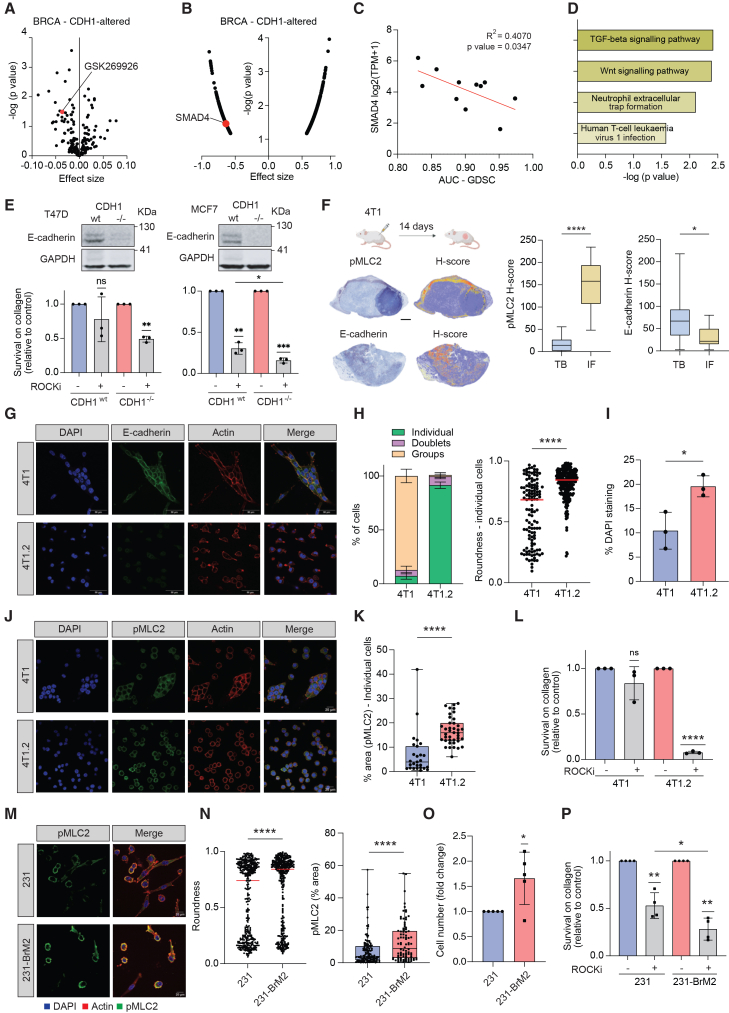
E-cadherin loss is associated with ROCK inhibitor response in epithelial cancer cells (A) Inhibitor correlation dependencies in CDH1-altered group from GDSC2 database in breast cancer cell lines. Negative effect size indicates higher sensitivity of CDH1-altered cell lines to inhibitors. Dots show independent inhibitors tested for sensitivity. (B) Gene correlation dependencies to ROCK inhibitor sensitivity in *CDH1*-altered breast cancer cell lines. Negative effect size indicates higher expression in sensitive samples. (C) SMAD4 expression correlation plot of CDH1-altered breast cancer cell lines to ROCK inhibitor GSK269962 AUC from GDSC2. Statistical test, simple linear regression. (D) KEGG pathway enrichment analysis of genes showing negative effect size from Figure 2C. (E) Western blot of T47D and MCF7 cells and their E-cadherin KO counterparts. Blotting for E-cadherin and GAPDH (top). Survival of T47D Cas9 and T47D CDH1^−/−^ pair of cell lines on collagen matrices upon addition of GSK269962 5 μM for 72 h (bottom left) and MCF7 Cas9 and MCF7 CDH1^−/−^ pair (bottom right). Bar plot shows mean value with whiskers showing SD. All points shown. Statistical tests, unpaired *t* test for cell growth; one sample *t* test for relative survival against control. *n* = 3 biological replicates. (F) Representative images from pMLC2 and E-cadherin staining in 4T1-derived tumors (left) and quantification of staining in Qupath comparing TB and IF regions of the tumors (right). Boxplots show median value and interquartile range with whiskers showing min to max values. Statistical test, unpaired *t* test. Scale bars, 1 mm. *n* = 13 tumors; TB and IF quantified within each tumor. (G) Immunofluorescence of 4T1 and 4T1.2 cells grown in thick collagen gels and stained for DAPI, E-cadherin, and phalloidin. Scale bars, 50 μM. (H) Quantification of individual cells and clusters (left) and roundness of individual cells from (G). Morphology quantification shows stacked bars mean value with SD. Dot plot shows all points with red line indicating mean value. Statistical test, unpaired *t* test. Three biological replicates; *n* = 121 cells (4T1) and 236 cells (4T1.2). (I) Cell growth of 4T1 cell line isogenic pair measured by % of DAPI staining on thick collagen gels. Bar plot shows mean value with whiskers showing SD. Statistical test, unpaired *t* test. *n* = 3 biological replicates. (J) Immunofluorescence of 4T1 and 4T1.2 cells stained for DAPI, pMLC2, and phalloidin. Same conditions as (G) Scale bars, 20 μM. (K) Quantification from immunofluorescence of pMLC2 intensity (% area) from (J). Box shows median value with interquartile range; whiskers show min to max values. Statistical test unpaired *t* test. Three biological replicates; *n* = 27 cells (4T1) and *n* = 40 cells (4T1.2). (L) Survival of 4T1 isogenic cell lines in thick collagen matrices upon addition of GSK269962 1 μM for 72 h. Bar plot shows mean value with whiskers showing SD. All points are shown. Statistical tests, unpaired *t* test for cell growth, one sample *t* test for relative survival against control. *n* = 3 biological replicates. (M) Immunofluorescence images of MDA-MB-231 and MDA-MB-231-BrM2 cells seeded on top of a thick collagen matrix and stained for DAPI (405), phalloidin (647), and pMLC2 (546). Scale bars, 20 μM. (N) Quantification of cell roundness (left) and pMLC2 (% intensity per area) (right) from 2M. Roundness, dot plot shows individual values. Statistical test, unpaired *t* test. pMLC2 quantification, box shows interquartile range; whiskers show min to max values. Statistical tests, unpaired *t* test. Seven biological replicates; *n* = 284 cells (231) and *n* = 376 cells (231-BrM2). (O) Quantification of cell growth in collagen of MDA-MB-231 and MDA-MB-231-BrM2. Box shows mean value with whiskers showing SD; all points shown. Statistical tests, one sample *t* test. *n* = 5 biological replicates. (P) Quantification of cell survival of MDA-MB-231 and MDA-MB-231-BrM2 cells untreated or treated with GSK269962 1 μM for 72 h from DAPI staining. Box shows mean value with whiskers showing SD. Statistical test, one sample *t* test for independent cell line survival vs. control and unpaired *t* test between the fold-change values of each cell line treated. *n* = 4 biological replicates. For (E), (F), (H), (I), (K), (L), (N), (O), and (P), ns = not significant, ∗*p* < 0.05, ∗∗*p* < 0.01, ∗∗∗*p* < 0.001, ∗∗∗∗*p* < 0.0001.

We further tested this observation in breast cancer models, a disease where E-cadherin mutation, copy number loss and/or loss of protein expression is relatively common.^47^^,^^48^ To do this, we separated breast cancer cell lines into *CDH1*-altered (genetic inactivation or loss of protein expression) and *CDH1*-unaltered cohorts. Using this classification, we found that *CDH1* alteration correlates with higher sensitivity to GSK269962 (Figure 2A; Figure S2C). Although the correlation between CDH1 alteration and GSK269962 sensitivity was clear, GSK269962 sensitivity still varied across the cohort of *CDH1*-altered breast cancer cell lines, suggesting that other features, in addition to *CDH1* status, might also predict GSK269962 response. To identify these other features, we assessed transcriptomic data from *CDH1*-altered breast cancer cell lines only, finding that increased SMAD4 expression in *CDH1*-altered cell lines correlated with increased ROCK inhibitor sensitivity (Figures 2B and 2C). Moreover, when performing pathway enrichment analysis, with the genes correlating with ROCK inhibitor sensitivity in the CDH1-altered cohort, we observed a positive correlation between ROCK inhibitor responses and enrichment in genes related to TGF-β and Wnt signaling (Figure 2D). This is in accordance with previous studies connecting high ROCK-Myosin II in amoeboid cancer cells to crosstalk with such signaling modules.^5^^,^^17^

To validate these predictions made using high-throughput drug screens (i.e., from the GDSC data), we formally tested whether loss of E-cadherin could drive sensitivity to ROCK inhibitors in breast cancer. To do this, we used previously described CDH1 wildtype and isogenic CDH1 knockout (KO) MCF7 breast cancer cells^49^ as well as generating (via CRISPR-Cas9 mutagenesis of *CDH1*) new CDH1 wildtype and isogenic CDH1 cells from ER^+^ T47D breast cancer cells. When we compared the response to ROCK inhibition in T47D cells, we found that the CDH1-KO cells were sensitive to ROCK inhibition, whereas the E-cadherin wild type cells were not (Figure 2E). Although E-cadherin wildtype MCF7 cells showed some intrinsic sensitivity to ROCK inhibition, MCF7 KO cells showed greater sensitivity (Figure 2E). Interestingly, MCF7 parental line had lower basal levels of E-cadherin when compared to the non-responder T47D parental line and therefore showed a stronger response to ROCK inhibitor (Figure 2E). These findings support E-cadherin loss as a potential marker of ROCK inhibitor sensitivity in breast cancer.

We next grew tumors from E-cadherin positive 4T1 cells and characterized two different regions in these tumors: the tumor body (TB) and the invasive front (IF). We observed that pMLC2 levels, indicative of active Myosin II, were increased in cells located at the IF (Figure 2F), correlating with decreased E-cadherin levels (Figure 2F) while no changes in CK8 were apparent (Figure S2D), suggesting an increase on amoeboid invasive features. These data suggest that within epithelial tumors there may be a proportion of cells undergoing an epithelial-to-amoeboid transition at the tumor border.

While the 4T1 TNBC cell line was derived from a spontaneous mammary tumor of a Balb/c mouse and can metastasize to the lung and lymph nodes, the 4T1.2 cell line is able to metastasize to the lung, lymph nodes, and bone,^50^ making it more metastatic. These two lines differed in their E-cadherin levels (Figure 2G; Figure S2E), cell individualization, roundness index (Figure 2H), pMLC2 levels (Figures 2J and 2K), and cell survival on collagen I (Figure 2I). These data show how 4T1 cells are largely epithelial with a small proportion of amoeboid cells, while 4T1.2 are mainly amoeboid and have lost epithelial features, similar to what we observed at the border of the tumor (Figure 2F). ROCK inhibitor (GSK269962) treatment revealed that the amoeboid isogenic counterpart, 4T1.2, was more responsive to ROCK inhibition than the more epithelial cell line, 4T1 (Figure 2L), with the reduced response in 4T1 cells most likely occurring in the small proportion of amoeboid cells present in this cell line.

Since we found Myosin II activity to be linked to ROCK inhibitor responses in E-cadherin-altered cell lines, we next investigated whether ROCK inhibitor sensitivity was exclusively E-cadherin-dependent or if intrinsic levels of myosin II activity could also play a role. To study this, we used the MDA-MB-231 isogenic model where both TNBC cell lines have lost E-cadherin as they have undergone EMT. The MDA-MB-231-BrM2 cell line was isolated from parental MDA-MB-231 after three rounds of brain metastasis formation.^51^ Again, the more metastatic cell line of the pair is more rounded (Figures 2M and 2N), displays increased pMLC2 levels (Figures 2M and 2N) and higher survival on a collagen I matrix (Figure 2O). Importantly, even though both cell lines responded to ROCK inhibition, MDA-MB-231-BrM2 cells were more sensitive than their less metastatic and less amoeboid counterpart (Figure 2P).

Overall, these data show that cancer cells that have undergone EMT and lost E-cadherin are more sensitive to ROCK inhibition, and within the EMT spectrum, amoeboid cancer cells are particularly sensitive to ROCK inhibitors. This suggests that both E-cadherin status and levels of Myosin II activity may be useful as biomarkers of ROCK inhibitor sensitivity in tumors of epithelial origin.

### NF-κB activity is associated with ROCK inhibitor responses in non-epithelial solid tumors

In cancer cells, E-cadherin expression can be lost due to hypermethylation of the *CDH1* promoter among other mechanisms.^52^ On the other hand, melanocytes, the cell-of-origin in melanoma, are derived from the neural crest, which undergoes EMT during development.^19^^,^^20^ Therefore, in melanoma, EMT gene expression has been associated with the acquisition of stem cell-like and invasive properties, rather than a canonical EMT.^17^

**Figure 3:**
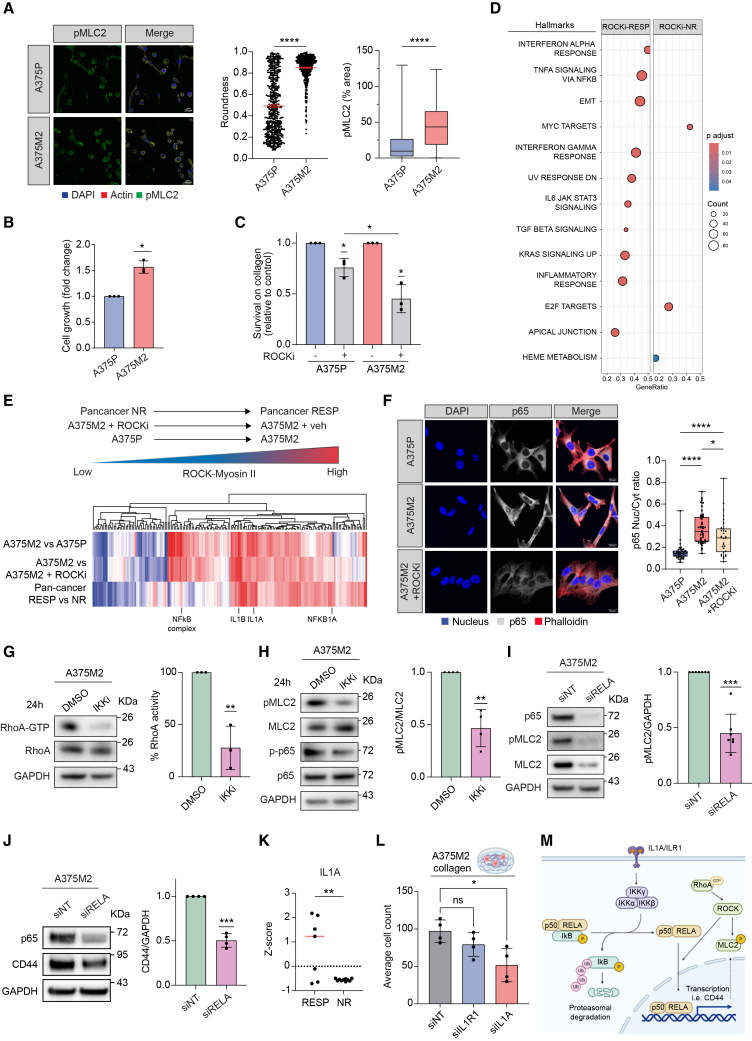
NFKB activity is associated with ROCK inhibitor responses in non-epithelial solid tumors (A) Immunofluorescence of A375P and A375M2 cells seeded on top of collagen I thick matrices and stained for pMLC2, phalloidin, and DAPI (left). Scale bars, 20 μM. Quantification of cell roundness in collagen (middle) and pMLC2 staining (right) from immunofluorescence. Dot plot shows all individual values with red bars showing mean value with SEM. Boxplot shows median value with interquartile range; whiskers show min to max values. *n* = 3 biological replicates. For roundness, *n* = 537 cells (A375P) and *n* = 500 cells (A375M2). For pMLC2 quantification, *n* = 275 cells (both cell lines). (B) Quantification of cell growth in collagen measured as fold change in cell number after 72 h between A375M2 and A375P cells. Bar plot shows mean value with whiskers showing SD. Statistical test, one sample *t* test. *n* = 3 biological replicates. (C) A375P and A375M2 cells growing on thick collagen I gels, untreated or treated with GSK269962 1 μM for 72 h stained with DAPI (left) and quantification (right). Bar plot shows mean value with whiskers showing SD. Statistical tests include one sample *t* test for cell growth and independent cell line survival vs. control and unpaired *t* test between the fold-change values of each cell line treated. *n* = 3 biological replicates. (D) Dot plot of upregulated terms in ROCK inhibitor-responder and non-responder melanoma cell lines using the Hallmarks database. (E) Schematic of the gene expression profiles comparison, including pan-cancer cell line ROCK inhibitor-sensitivity comparison and published transcriptomes of A375P, A375M2, and A375M2 treated with different ROCK inhibitors (top) and ingenuity pathway analysis upstream analysis shown in heatmap for the three comparisons. Highlighted are the members of the NF-κB signaling pathway family (bottom). (F) Immunofluorescence of A375P, A375M2, and A375M2 + ROCKi stained for DAPI and p65 (RELA) (left) and quantification of nuclear/cytoplasmatic ratio of p65 (right). Box shows median value with interquartile range; whiskers show min to max values. Statistical test, one-way ANOVA. Scale bars, 20 μM. *n* = 3 biological replicates. For quantification, *n* = 94 cells (A375P); *n* = 90 cells (A375M2); and *n* = 81 cells (A375M2 + ROCKi). (G) Western Blot for RhoA pulldown of A375M2 cells treated with DMSO or IKKi LY2409881 for 24 h 1 μM. Blotting for RhoA-GTP (pulldown fraction), total RhoA and GAPDH (left) and quantification measuring RhoA-GTP signal/total RhoA. Bar plot shows mean value with whiskers showing SD. Statistical test one sample *t* test (right). *n* = 3 biological replicates. (H) Western Blot for pMLC2 (Ser18/Thr19), MLC2, p-p65 (Ser536), p65, and GAPDH for same conditions as (G) (left) and quantification for pMLC2/MLC2. Bar plot shows mean value with whiskers showing SD. Statistical test one sample *t* test. *n* = 4 biological replicates. (I) Western blot of A375M2 cells transfected with siNT or siRELA (p65). Blotting for p65, pMLC2 (Ser18/Thr19), MLC2, and GAPDH (left) and quantification of pMLC2/GAPDH. Bar plot shows mean value with whiskers showing SD. Statistical test one sample *t* test. *n* = 7 biological replicates. (J) Western blot of A375M2 cells transfected with siNT or siRELA (p65). Blotting for p65, CD44, and GAPDH (left) and quantification of CD44/GAPDH. Bar plot shows mean value with whiskers showing SD. Statistical test one sample *t* test. *n* = 4 biological replicates. (K) Comparison of *Z* score expression between ROCK inhibitor-responder and ROCK inhibitor-non-responder melanoma cell lines for *IL1A*. Statistical test unpaired *t* test. *n* = 7 cell lines (responders) and *n* = 10 cell lines (non-responders). Red bar shows median value. (L) Cell survival of A375M2 cells measured by cell count on collagen 72 h after transfection with siIL1A and siIL1R1. Bar plot shows mean value with whiskers showing SD. Statistical test one-way ANOVA comparing the mean of each column to the mean of the control column (siNT). *n* = 4 biological replicates. (M) Schematic of ROCK-Myosin II and NF-κB signaling pathway crosstalk in A375M2 cells. Schematic created with Biorender.com. For (A), (B), (C), (F), (G), (H), (I), (J), (K), and (L), ns = not significant, ∗*p* < 0.05, ∗∗*p* < 0.01, ∗∗∗*p* < 0.001, ∗∗∗∗*p* < 0.0001.

We next used isogenic pairs of melanoma cell lines: A375P and A375M2. A375M2 cells were isolated from A375P cells after undergoing three rounds of lung colonization and have been extensively characterized to have higher metastatic potential as well as high Rho-ROCK-myosin II activity.^4^^,^^5^^,^^13^^,^^53^^,^^54^ A375M2 were more rounded (Figure 3A), had higher myosin II activity (Figure 3A) and survived better on collagen I (Figure 3B). Importantly, A375M2 were more sensitive to ROCK inhibition than A375P (Figure 3C).

GSEA analysis of GDSC melanoma cell lines with differential ROCK inhibitor response led to the conclusion that EMT and inflammatory response-related genes were highly expressed in ROCK inhibitor responder melanoma cell lines (Figure 3D; Figure S3A), as seen at pan-cancer level (Figure 1). In addition, NF-κB signaling was among the top upregulated processes in ROCK inhibitor responder cell lines (Figure 3D; Figure S3A) compared to non-responders. We next hypothesized that amoeboid cancer cell gene signatures could be used to define biomarkers of ROCK inhibitor sensitivity. For that, we integrated our *in silico* pan-cancer analysis of ROCK inhibitor sensitivity genes with a gene expression dataset on A375M2 vs. A375P and A375M2 vs. A375M2 treated with ROCK or myosin II inhibitors (Figure 3E).^4^ Importantly, integration of these data showed that both ROCK inhibitor pan-cancer response gene sets and amoeboid melanoma gene sets share, among others, significant upregulation of genes related to NF-κB signaling, including NF-κB complex, *IL1A*, *IL1B*, and *NFKBIA* (Figure 3E).

NF-κB signaling is a known regulator of immune responses and inflammation.^55^ In cancer, NF-κB signaling has been described to aid in cancer cell survival by impairing apoptosis.^56^^,^^57^ On the other hand, NF-κB is upregulated in amoeboid melanoma cells, supporting an immunomodulatory secretome.^13^ To investigate the possible cell intrinsic regulation of NF-κB and cell survival in ROCK inhibitor responder cells, we first measured p65/RELA nuclear/cytoplasmic (N/C) ratios. A375M2 cells had higher N/C ratios compared to A375P cells and that ratio was reduced after ROCK inhibitor treatment (Figure 3F). These data show that in melanoma, ROCK controls N/C shuttling of NF-κB. Importantly, treatment with IKK inhibitor LY2409881 reduced both RhoA activity (Figure 3G) and downstream MLC2 phosphorylation (Figure 3H). In addition, reducing p65/RELA levels using RNAi resulted in both decreased MLC2 phosphorylation and total MLC2 protein levels (Figure 3I; Figure S3B). CD44 upregulation has been linked to loss of differentiated features or an increase in stemness,^58^ while we confirmed that p65/RELA reduction resulted in decreased CD44 (Figure 3J; Figure S3C). These data show that while ROCK regulates NF-κB localization, NF-κB signaling regulates Rho-ROCK-myosin II activity and de-differentiation, establishing a feedback loop.

We next argued that if NF-κB signaling is upregulated in amoeboid cancer cells with high Rho-ROCK signaling, they may also rely on this pathway for their survival. We evaluated whether the expression of any NF-κB signaling component was differentially regulated in ROCK inhibitor responder melanoma cell lines and confirmed that *IL1A* was significantly upregulated (Figure 3K; Figure S3D). Depletion of *IL1A* resulted in decreased survival of cancer cells growing on collagen I, while *IL1A* or *IL1R1* decreased cell survival in tissue culture conditions (Figure 3L; Figures S3E–S3G).

Overall, these data suggest high NF-κB activity is a further adaptation of amoeboid melanoma cells with high ROCK activity, resulting in a positive feedback loop between these two pathways (Figure 3M). As a result, ROCK inhibitor responder cells rely on IL1A-NFκB pathway, which drives their survival advantage and de-differentiated characteristics.

### ROCK and NF-κB crosstalk *in vivo*

**Figure 4:**
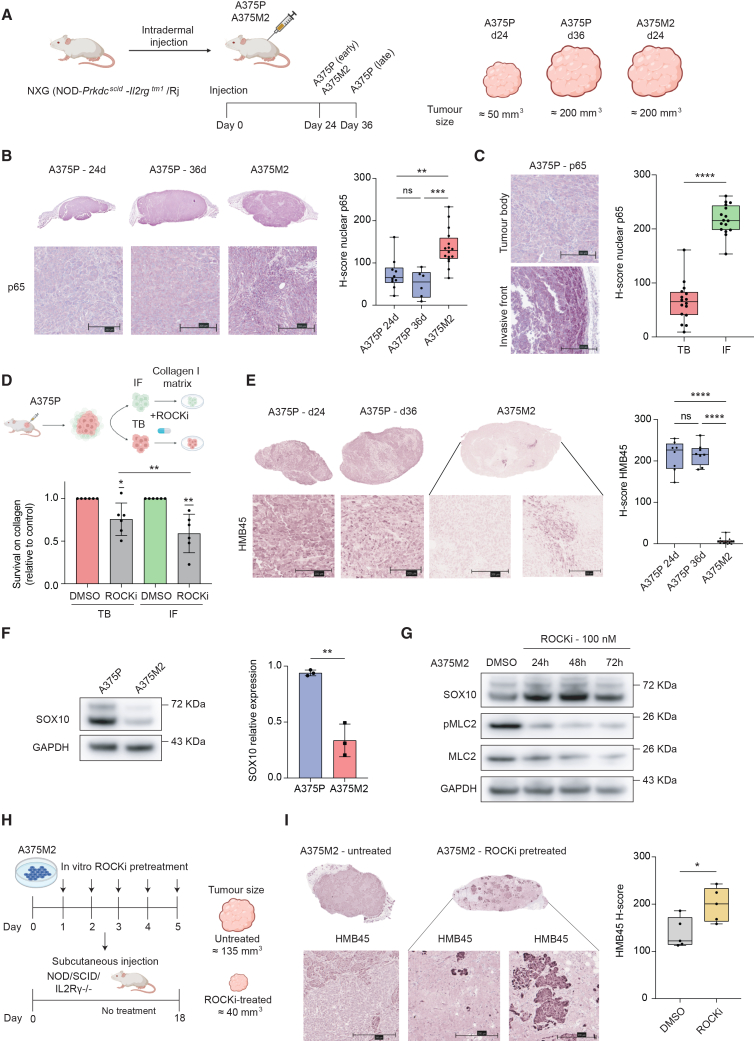
ROCK and NFκB crosstalk *in vivo* (A) Schematic of the *in vivo* model of A375 cell line isogenic pair. NXG mice were intradermally injected with A375P or A375M2 cells. Tumors grew for 24 days (A375M2) or 24 and 36 days (A375P) (left) and tumor volume of A375P-derived tumors or A375M2-derived tumors (right). Adapted from Jung-Garcia et al.^59^ Schematic created with Biorender.com. (B) Representative images from tumors derived from A375P at two different timepoints (24 and 36 days) and A375M2 after 24 days stained for p65 (left) and quantification of the percentage of p65 nuclear intensity in the TB (right). Boxplot shows median value and interquartile range with whiskers showing mean to max. Statistical test ordinary one-way ANOVA multiple comparisons comparing the mean of each column against the mean of every other column. Scale bars, 200 μM. *n* = 8 tumors (A375P d24), *n* = 8 tumors (A375P d36), and *n* = 16 tumors (A375M2). (C) Representative images of A375P stained for p65 from the TB and the IF of the tumors (left) and quantification of the percentage of p65 nuclear staining from (right). Boxplot shows median value and interquartile range with whiskers showing mean to max. Statistical test unpaired *t* test. Scale bars, 200 μM. *n* = 16 tumors; TB and IF quantified within each tumor. (D) Schematic showing TB and IF cell separation after extraction of subcutaneously injected A375P-derived tumors (left) and relative response to GSK269962 ROCK inhibition in these cells seeded on top of a collagen type I matrix (right). Data was normalized to DMSO-treated controls. Bar plot shows mean value with whiskers showing SD. All points are shown. Statistical test, one sample *t* test comparing the values to a fixed value of 1 and paired *t* test comparing ROCKi-treated conditions. *n* = 6 biological replicates. Schematic created with Biorender.com. (E) Representative images from tumors derived from A375P at two different timepoints (24 and 36 days) and A375M2 after 24 days stained for HMB45 differentiation marker (left) and quantification of the percentage of HMB45 H score (right). Boxplot shows median value and interquartile range with whiskers showing mean to max. Statistical test ordinary one-way ANOVA multiple comparisons comparing the mean of each column against the mean of every other column. Scale bars, 200 μM. *n* = 8 tumors (A375P d24), *n* = 8 tumors (A375P d36), and *n* = 16 tumors (A375M2). (F) Western blot of A375P and A375M2 cells for SOX10 melanoma differentiation marker and GAPDH (left) and quantification of SOX10 levels (right). Bar plot shows mean value with whiskers showing SD Statistical test unpaired *t* test. *n* = 3 biological replicates. (G) Western blot of A375M2 cells treated with 100 nM of ROCK inhibitor GSK269962 for 24, 48, and 72 h. Blotting for SOX10, pMLC2 (Ser18/Thr19), MLC2, and GAPDH. *n* = 3 biological replicates. (H) Schematic of *in vivo* experiment with ROCK inhibitor H1152 pretreated A375M2 cells for five days, subcutaneously injected in SCID mice for 18 days. Adapted from Rodriguez-Hernandez et al.^17^ Schematic created with Biorender.com. (I) Representative images of A375M2-derived tumors untreated or pretreated with ROCK inhibitor H1152 stained for HMB45 melanoma differentiation marker (left) and quantification of HMB45 H score in pretreated and untreated tumors (right). Boxplot shows median value and interquartile range with whiskers showing mean to max. Statistical test unpaired *t* test. Scale bars, 200 μM. *n* = 5 tumors (DMSO) and *n* = 5 tumors (ROCKi). For (B), (C), (D), (E), (F), (G), and (I), ns = not significant, ∗*p* < 0.05, ∗∗*p* < 0.01, ∗∗∗*p* < 0.001, ∗∗∗∗*p* < 0.0001.

It was important to validate our observations *in vivo*. Orthotopic injection of A375P and A375M2 cells in the dermis of NXG mice led to establishment of tumors, where A375M2-derived tumors grew faster and invaded more;^59^ hence, A375P-derived tumors were obtained at two different timepoints to match either the A375M2 collection time (24 days) or when they reached a similar tumor size to A375M2-derived tumors (36 days) (Figure 4A). As seen *in vitro* (Figure 3F), nuclear p65 levels were higher *in vivo* in A375M2-derived tumors, compared to A375P-derived timepoints (Figure 4B). Since amoeboid cancer cells have been located at the IF of tumors,^13^^,^^17^^,^^59^ we characterized the TB and IF of tumors. Nuclear p65 signal was higher in cells located at the IF of tumors in both conditions and timepoints (Figures 4C; Figure S4A), suggesting that invasion and pro-inflammatory/pro-survival signaling are linked. We next isolated cells from these two areas and grew them *ex vivo* in collagen I matrices (Figure 4D). While they were both sensitive to ROCK inhibition, cells isolated from the IF were more sensitive to ROCK inhibition than those from the TB (Figure 4D).

We next stained tumors for commonly used melanoma differentiation markers, HMB45 and SOX10.^60^^,^^61^ We observed lower levels of these markers in A375M2-derived tumors, where their expression was restricted to specific regions within the center of the tumor (Figure 4E; Figure S4B). SOX10 levels were also lower in A375P-derived tumors at the 36-day timepoint when compared to 24 days, suggesting that these tumors become more de-differentiated as they grow larger in size (Figure S4B). SOX10 levels were lower in A375M2 cells compared to A375P cells (Figure 4F) when grown *ex vivo*, suggesting that during the *in vivo* generation of the highly metastatic A375M2 cell line, a process of de-differentiation took place. Importantly, upon treating these cells with a low dose of ROCK inhibitor for as little as 24 h, an increase in the expression of melanoma differentiation marker SOX10 was detected (Figure 4G). Next, we hypothesized that cancer cells that grow *in vivo* with lower actomyosin from the start, may develop tumors with more differentiated cancer cells later on, even if they recover their actomyosin levels. To assess this, we pre-treated A375M2 cells with a ROCK inhibitor *ex vivo* for five days followed by injection of viable/alive cancer cells in mice in equal numbers (Figure 4H). By pre-treating the cancer cells, we can assess the effects of the ROCK inhibition only in the cancer cells and not the stroma. Previous work showed that 18 days post-injection, ROCK inhibitor pre-treated A375M2-derived tumors showed over 60% reduction in tumor weight.^17^ ROCK inhibition in this setting led to decreased viability and proliferation, as well as reduced capacity for tumor initiation and growth *in vivo*.^17^ We analyzed sections from these tumors and observed that ROCK inhibitor pre-treated tumors showed higher levels of HMB45, indicating that a ROCK inhibitor treatment reduced growth in a large number of cells,^17^ but the remaining surviving cells retained high levels of differentiation-related markers (Figure 4I), suggesting that ROCK suppresses cancer cell differentiation in a cell autonomous manner.

Overall, these results show how cells with high NF-κB signaling are located in the invasive areas of tumors while we find a negative association between ROCK activity and differentiation markers *in vivo*.

### Characterization of AML response to ROCK inhibitors

**Figure 5:**
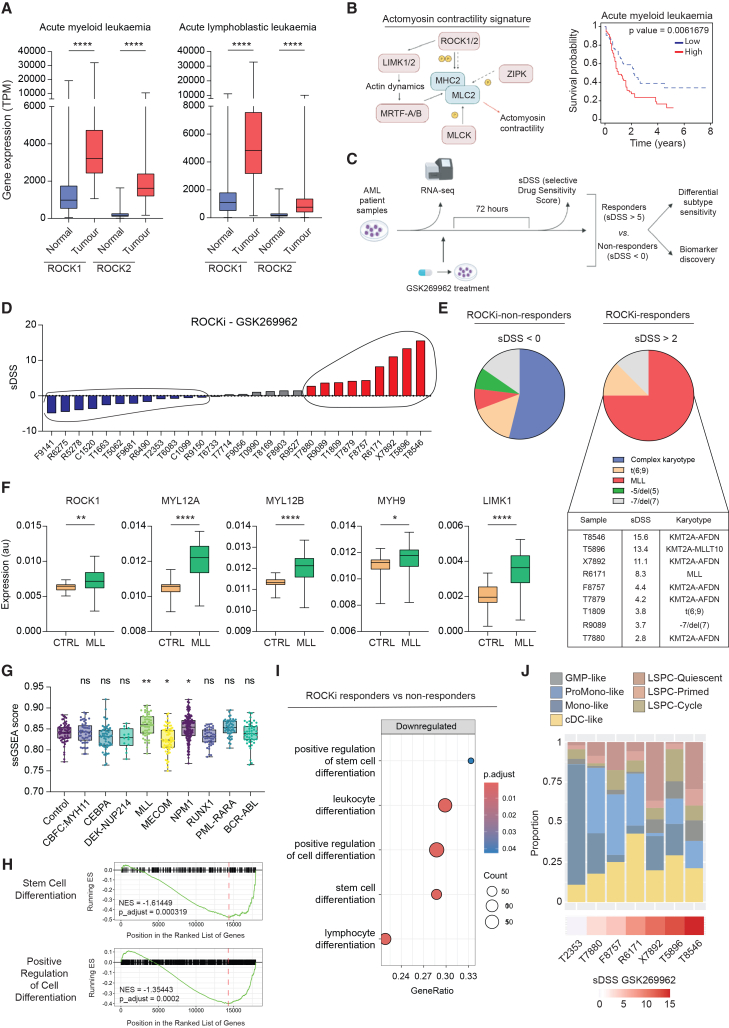
Characterization of AML response to ROCK inhibitors (A) Gene expression of ROCK1 and ROCK2 comparing tumor vs. normal cells from TCGA, TARGET and GTEx databases. Obtained from TNMplot.^62^ Boxes show median value with interquartile range; whiskers show min to max values. For AML, *n* = 407 samples (normal); *n* = 151 samples (tumor). For ALL, *n* = 407 samples (normal); *n* = 122 samples (tumor). (B) 12-gene actomyosin contractility signature from^12^ (left) and overall survival Kaplan-Meier plot dividing patients by median signature value. Statistical test, log-rank test. Schematic created with Biorender.com. (C) Schematic of primary AML poor-risk patient samples’ drug screening from Casado et al.^63^ Bulk RNA-seq of primary AML samples was performed prior to the addition of any inhibitor. Samples were screened against an array of inhibitors for 72 h and separated into responder (sDSS > 2 or sDSS > 5) and non-responders (sDSS < 0) for each inhibitor. Schematic created with Biorender.com. (D) Distribution of drug response to ROCK inhibitor GSK269962 from primary AML samples. (E) AML karyotype separation of ROCK inhibitor-responder and non-responder samples (top) and karyotype breakdown of ROCK inhibitor-responder samples with an sDSS > 2 (bottom). (F) Normalized mRNA expression of ROCK1, MYL12A, MYL12B, MYH9, and LIMK1 in control samples and MLL-r samples obtained from TARGET-AML (dbGaP Accession, phs000465.v23.p8) and GEO omnibus under accession numbers GSE118963, GSE107490, GSE72061, GSE117994, GSE104406, and GSE113182. Boxplots show median value with interquartile range, and whiskers show min to max values. Statistical test, unpaired *t* test. *n* = 45 samples (control); *n* = 60 samples (MLL). (G) ssGSEA score of 5-gene signature from (F) in AML validation cohort separated by karyotype. Boxplots show median value with interquartile range; whiskers show min to max values. All points shown. Statistical test, one way ANOVA comparing the mean of each column to control. *n* = 64 samples (control); *n* = 50 samples (CBFC:MYH11), *n* = 59 samples (CEBPA), *n* = 14 samples (DEK:NUP214), *n* = 52 samples (MLL), *n* = 67 samples (MECOM), *n* = 163 samples (NPM1), *n* = 44 samples (RUNX1), *n* = 52 samples (PML:PARA), and *n* = 48 samples (BRC-ABL). (H) Differentiation-related GSEA plots from ROCK inhibitor-responders vs. ROCK inhibitor-non-responders AML samples using GOBP. NES and *p* adjust shown. (I) Dot plot of selected differentiation-related genes from a GSEA comparing ROCK inhibitor-responder and ROCK-non-responders using GOBP. (J) Deconvolution analysis using single-cell references from leukemia stem, progenitor and mature cell types from bulk RNA-seq datasets as described by Zeng et al.^64^ using the MLL-r samples from the screening. For (A), (F), and (G), ns = not significant, ∗*p* < 0.05, ∗∗*p* < 0.01, ∗∗∗*p* < 0.001, ∗∗∗∗*p* < 0.0001.

Our data in Figure 1 suggested that hematological malignancies were high responders to ROCK inhibition. To gain unbiased insight into the activation of the ROCK-Myosin II pathway in hematological malignancies, we measured pMLC2 levels, indicative of high ROCK pathway activity, in a tumor microarray, including a variety of leukemias and lymphomas. Increased levels of MLC2 phosphorylation were observed in most samples when compared to healthy T cell control (Figures S5A and S5B), while AML showed consistently high levels of myosin II activation (Figures S5A and S5B). Using TCGA and TARGET data, we confirmed that mRNA levels of both ROCK isoforms were upregulated in cancer when compared to normal tissue (Figure 5A) in both AML and acute lymphoblastic leukemia (ALL). We next evaluated a 12-gene signature comprised genes directly contributing to actomyosin contractility regulation^12^ (Figure 5B) and found high expression levels correlated with worse survival probability in AML patients (Figure 5B) suggesting the potential role of ROCK-Myosin II in driving aggressive disease.

In leukemia, translocations and chromosome duplications are common originating events, rather than the steady accumulation of mutations found in solid tumors. Previous studies have shown that ROCK inhibition could be beneficial in the treatment of AML.^31^^,^^32^^,^^65^^,^^66^ However, these studies did not identify a particular AML karyotype with increased sensitivity. To answer this question, we used a drug screening on primary AML adverse-risk patient samples with different karyotypes,^63^ where drug sensitivity is calculated taking into account the effectiveness of the drug in targeting cancer cells and the lack of adverse effects in healthy bone marrow (Figure 5C). We categorized samples as non-responders or responders to ROCK inhibitor GSK269962 based on their selective drug sensitivity score (Figure 5D) and observed that the mixed lineage leukemia (*MLL*-rearranged; MLL-r) karyotype (samples with a *KMT2A* translocation) was highly responsive to ROCK inhibitor (Figure 5E). KMT2A is a gene frequently involved in chromosomal translocations driving acute myeloid leukemia, resulting in aggressive, stem-like and poorly differentiated leukemia states.^67^^,^^68^ Interestingly, MLL1 has previously been linked to metastasis through a ROCK-pMLC2 axis in breast cancer.^69^ Moreover, MLL translocations have common fusion partners that differ in the biology of the disease and its prognosis.^67^ Within ROCK inhibitor responder MLL samples, those with *KMT2A-AFDN* and *KMT2A-MLLT10* rearrangements were the most responsive to the ROCK inhibitor (Figure 5E). This subset of samples was previously defined as part of a specific subgroup, which is highly sensitive to inhibition of mitotic kinases or genotoxic drugs and shows increased DOT1L phosphorylation and CDK1 activity.^63^ In addition, we evaluated the expression of the 12 genes comprising the ROCK-Myosin II signature between control and MLL-rearranged karyotypes from different publicly available databases and observed that five out of the 12 genes were significantly overexpressed in MLL-r patients (Figure 5F). Specifically, *ROCK1*, *MYL12 A/B*, genes encoding for MLC2, *MYH9*, the main myosin heavy chain, and *LIMK1* (Figure 5F). This shows how MLL-r AML upregulates both ROCK and Myosin II machinery at the mRNA level. Importantly, this was further confirmed in an independent AML patient dataset where higher expression of a signature derived from these five genes is significantly upregulated in MLL and NPM1-AML samples, the latter present in 20%–30% of AML cases,^70^ when compared to control (Figure 5G).

Importantly, MLL rearrangements lead to a deregulation of transcription and the activation of genes related to early hematopoiesis^67^ as well as having a role in hematopoietic stem cell self-renewal.^71^ We next analyzed the transcriptomes of ROCK inhibitor responder samples, predominantly those with *KMT2A-AFDN* and *KMT2A-MLLT10* rearrangements. GSEA analysis in ROCK inhibitor responder samples compared to non-responders showed enrichment in genes related to cell cycle, such as G2M checkpoint and E2F targets, as well as other processes related to metabolism, such as fatty acid metabolism and oxidative phosphorylation (Figure S5C). In addition, terms involved in processes related to cell differentiation were downregulated in ROCK inhibitor responder samples (Figures 5H and 5I), similar to the previous findings in solid tumors. Importantly, by performing a deconvolution analysis, using single-cell references from leukemia stem, progenitor and mature cell types from bulk RNA-seq datasets,^64^ we determined that ROCK inhibitor sensitivity in MLL samples is correlated with a decrease in pro-monocytic-like and monocytic-like populations and an increase in the leukemia stem and progenitor cells (LSPCs) compartment, characterized by being more stem-like and less differentiated (Figure 5J). Moreover, Rho GTPase signaling and Myosin II binding-related genes were increased in responder samples, suggesting higher levels of Rho-ROCK-myosin II could be a biomarker of ROCK inhibitor response (Figures S5D and S5E), as observed in solid tumors.

Overall, these results suggest that ROCK is highly upregulated in AML and that higher expression of a ROCK-actomyosin contractility signature correlates with worse prognosis. In addition, within a subset of adverse risk patients, MLL rearrangements correlate with stronger responses to ROCK inhibition.

### ROCK inhibition has cytostatic and cytotoxic effects in AML cells

**Figure 6:**
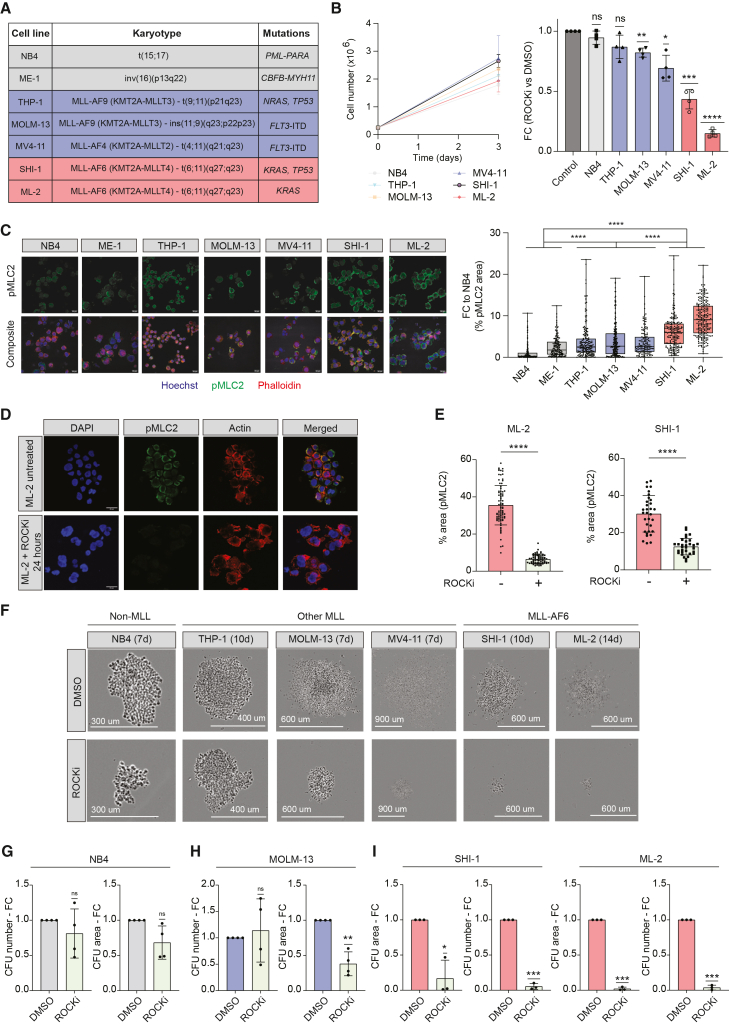
ROCK inhibition has cytostatic and cytotoxic effects in AML cells (A) Panel of AML cell lines including the seven different AML cell lines used for *in silico* and *in vitro* studies of ROCK inhibitor-sensitivity, including their karyotype and described driver mutations. (B) Cell proliferation in culture of a panel of six AML cell lines including NB4, THP-1, MOLM-13, MV4-11, ML-2 and SHI-1. Cells were seeded at a concentration of 0.5 × 10^6^ and counted after 72 h in culture (left) and bar plot of fold change in cell number of the AML panel of cell lines between low-dose treatment of 0.5 μM GSK269962 and DMSO control independent to each cell line (right). Bar plot shows mean value with whiskers showing SD. Statistical test ordinary one sample *t* test. *n* = 4 biological replicates. (C) Immunofluorescence images of ME-1, NB4, THP-1, MV4-11, MOLM-13, SHI-1, and ML-2 cells for DAPI (405), pMLC2 Ser19 (488), and phalloidin/actin (546) (left) and quantification of the levels of cortical pMLC2 by area (right) as a fold change to NB4 mean by experiment. Boxplot shows median expression with interquartile range. Whiskers show mean to max values. n = 3–6 biological replicates; *n* = 148 cells (ME-1); *n* = 181 cells (NB4); *n* = 187 cells (THP-1); *n* = 177 cells (MOLM-13); *n* = 174 cells (MV4-11); *n* = 192 cells (SHI-1); *n* = 231 cells (ML-2). Statistical test one-way ANOVA comparing the mean of each column against the mean of every other column, performed as non-MLL (NB4 and ME1; *n* = 329 cells) vs. other MLL (THP-1, MOLM-13 and MV4-11; *n* = 538 cells) vs. MLL-AF6 (SHI-1 and ML-2; *n* = 423 cells). Scale bars, 20 μM. (D) Immunofluorescence images of ML-2 untreated or treated with 100 nM of ROCK inhibitor GSK269962 for 24 h. Stained for DAPI (405), pMLC2 Ser 19 (546), and phalloidin/actin (647). Scale bars, 20 μM. (E) Quantification of the levels of cortical pMLC2 by area of ML-2 and SHI-1. Scatter dot plot shows mean value with whiskers showing SD. Statistical test, unpaired *t* test. *n* = 3 biological replicates; For ML-2, *n* = 85 cells (untreated); *n* = 72 cells (ROCKi-treated). For SHI-1, *n* = 31 cells (untreated); *n* = 31 cells (ROCKi-treated). (F) Images show colonies of AML cells in methylcellulose-based media of six cell lines from the AML panel after 7–14 days of seeding. *n* = 4 biological replicates (NB-4, THP-1, MOLM-13 and MV4-11) and *n* = 3 biological replicates (SHI-1 and ML-2). (G) CFU quantification of colony number and colony area in GSK269962 ROCK inhibitor-treated and untreated conditions for NB4. Bar plot shows mean value with whiskers showing SD. Statistical test, one sample *t* test. *n* = 4 biological replicates. (H) CFU quantification of colony number and colony area in GSK269962 ROCK inhibitor-treated and untreated conditions for MOLM-13. Bar plot shows mean value with whiskers showing SD. Statistical test, one sample *t* test. *n* = 4 biological replicates. (I) CFU quantification of colony number and colony area in GSK269962 ROCK inhibitor-treated and untreated conditions for SHI-1 and ML-2. Bar plot shows mean value with whiskers showing SD. Statistical test, one sample *t* test. *n* = 3 biological replicates. For (B), (C), (E), (G), (H), and (I), ns = not significant, ∗*p* < 0.05, ∗∗*p* < 0.01, ∗∗∗*p* < 0.001, ∗∗∗∗*p* < 0.0001.

We next challenged an array of different AML cell lines for ROCK inhibitor sensitivity (Figure 6A). Using *Z* score values from the GDSC database, where a low value corresponds with a low IC_50_ to the inhibitor, we found that NB4 and ME-1 cells (non MLL-r) show no preferential sensitivity to ROCK inhibitors GSK269962, GSK429286, and Y-39983 (Figure S6A). MV4-11, MOLM-13, and THP-1 cell lines (MLL karyotypes other than MLL-AFDN-r) showed high sensitivity to the three different ROCK inhibitors (Figure S6A). ML-2, the only available MLL-AFDN-r cell line in the screening, showed higher sensitivity to all ROCK inhibitors and, importantly, ROCK inhibitor GSK429286 was ranked as the most effective drug (out of 358 available compounds in the GDSC screening) in this cell line (Figure S6A).

We validated this publicly available data using the same collection of five AML cell lines, plus SHI-1 as an additional MLL-AFDN-r cell line (Figure 6A). Proliferation rates among cell lines were similar (Figure 6B). While ROCK inhibition had no effect on the growth of NB4 or THP-1 cells, it impaired the growth of MOLM-13 and MV4-11 cells and had the strongest effect on SHI-1 and ML-2 cells in this assay (Figure 6B). Importantly, the highest pMLC2 levels and amoeboid features were observed in MLL-AFDN-r cells (Figure 6C). Myosin II activity in SHI-1 and ML-2 cells was ROCK-dependent as shown by the strong decrease in pMLC2 levels after treating with low ROCK inhibitor concentrations for 4 h (Figure S6B) or 24 h (Figures 6D and 6E; Figure S6D). Longer treatments (six days) with higher doses (0.5 μM ROCK inhibitor) resulted in higher cytotoxic effects in ML-2 and SHI-1 cells (Figure S6C). Overall, these data suggest that at least in this limited set of cancer cells, higher levels of Rho-ROCK-Myosin II signaling correlate with ROCK inhibitor responses.

Finally, since ROCK inhibitor response could be associated with stemness, we performed colony forming units (CFU) assays. A low dose of ROCK inhibitor (100 nM) did not impair colony number or colony area in NB4 cells (Figures 6F and 6G). On the other hand, ROCK inhibitor treatment reduced colony area, but not clonogenic potential, of other MLL-rearranged cells suggesting a cytostatic effect (Figures 6F and 6H). Finally, low dose ROCK inhibitor treatment resulted in decreased colony forming potential and reduced colony area in ML-2 and SHI-1 cells (Figures 6F and 6I). These data suggest that while ROCK inhibition has a cytostatic effect in MLL-r cells, it blocks stemness properties and results in cytotoxicity in MLL-AFDN-rearranged cells. Therefore MLL-AFDN-r could be a biomarker for extreme ROCK inhibitor sensitivity in AML patients.

## Discussion

Increased levels of Rho-ROCK-Myosin II signaling activity have been reported in a wide variety of cancers, where high levels correlate with increased invasion, migration, formation of metastases, and ultimately worse clinical outcome.^4^^,^^5^^,^^12^^,^^13^^,^^17^^,^^18^^,^^34^^,^^54^^,^^72^^,^^73^^,^^74^^,^^75^^,^^76^^,^^77^^,^^78^^,^^79^ Inhibitors targeting this pathway have been proposed for cancer treatment in different preclinical models. Nevertheless, their translation to a clinical setting has often been limited to hypertension or glaucoma,^25^^,^^26^ partly due to the hypotensive side effects experienced by patients treated with ROCK inhibitors, mainly attributed to the inhibition of both ROCK isoforms.^26^^,^^80^^,^^81^ In addition, other factors have contributed to slow translation to the clinic: lack of understanding of isoform-specific functions, poorly characterized downstream effectors, the use of non-selective and weak inhibitors, such as Y27632, and most importantly, lack of response biomarkers.^25^ The development of new generation ROCK inhibitors, such as GSK269962 used in this study, with lower IC_50_ values for ROCK1 and ROCK2 and reduced off-target profiles, offers new possibilities to study ROCK-dependent processes regulating cell survival with improved specificity.

More recently, the development of ROCK2-specific inhibitors have successfully reduced the observed side effects in the clinic and have been approved for the treatment of GvHD.^25^^,^^27^^,^^82^ Moreover, ROCK2-specific inhibitors are currently in clinical trials for cancer in patients with relapsed/refractory multiple myeloma (MM) (CTID, NCT06105554). ROCK1 and ROCK2, while structurally related and often considered functionally redundant, do exhibit both overlapping and divergent biological roles. While both isoforms have been described to be redundant in maintaining actomyosin contractility and cell proliferation,^7^ they also have distinct roles for other biological functions such as a more predominant role of ROCK2 in the regulation of cell detachment, differentiation and inflammation.^83^^,^^84^ For this reason, we focused this study on pan-ROCK inhibitors, in order to avoid potential redundant actions of both isoforms.

There are nearly 200 different available ROCK inhibitors,^26^ including (a) small molecule inhibitors based on isoquinoline, indazole or pyridine scaffolds, (b) soft ROCK inhibitors designed to act locally and then become inactivated into non-toxic forms once in circulation, or (c) dual-function inhibitors that inhibit ROCK and other targets. These latest generation ROCK inhibitors should be combined with the knowledge we provide in the current work defining biomarkers of response.

While some biomarkers of sensitivity are tumor-type dependent, others are shared across different cancer types, such as de-differentiation. Cancer cells that have not undergone EMT or are in a more epithelial-like differentiated state may use additional signaling pathways to sustain survival and growth that are not ROCK-dependent. While cancer cells that are in a more epithelial state are not responsive *in vitro* to ROCK inhibition based on their survival, previous studies show that ROCK inhibition impacts other important non-cell autonomous functions, including the alteration of their secretome,^37^ with changes having a long-term impact on tumor growth. Pre-treatment with ROCK inhibitors leads to the initial establishment of an “atypical” tumor microenvironment characterized by the infiltration of anti-tumoral macrophages and a consequential reduction in long-term *in vivo* growth.^37^

On the other hand, cancer cells that have undergone EMT or cancer cells with pathogenic E-cadherin mutations, may rely on high ROCK-Myosin II contractility not only to support their cell migration^28^^,^^85^ but also cell survival. Within the group of cancer cells with E-cadherin-dysfunction, higher sensitivity to ROCK inhibitors is associated with high TGF-β signaling, while this signaling cascade aids amoeboid cancer cell dissemination.^5^ Furthermore, loss of E-cadherin, in conjunction with p53 mutations,leads to survival and tumor growth through a p120-Rho-ROCK axis in lobular breast cancer.^8^ These mechanisms could also contribute to ROCKi sensitivity in epithelial cancer cells. On the other hand, ROCK signaling has been linked to IL-1α secretion^13^, while NF-κB regulates IL-6 secretion.^86^^,^^87^^,^^88^ Moreover, cytokine signaling through the IL6-JAK/STAT3 axis activates ROCK signaling and sustains actomyosin contractility.^4^ These connections could contribute to the described regulatory crosstalk between ROCK and NF-κB.

We found that in non-epithelial tumors, such as melanoma, RhoA/ROCK, and NF-κB signaling establish a positive feedback loop. Rho-ROCK-Myosin II supports efficient amoeboid type of migration and higher levels of NF-κB, while both pathways establish a crosstalk to control cancer cell survival. Since amoeboid cancer cells contribute to the aggressiveness of the disease,^2^ ROCK inhibition is an attractive strategy to reduce both cancer cell survival and dissemination.^25^^,^^26^^,^^89^ We hypothesize that cancer cells at the borders of tumors may respond particularly well to these approaches. In addition, several subtypes of hematological malignancies, including activated B-cell diffuse large B cell lymphoma^90^^,^^91^ and some cases of MM^92^ have mutations in different components of the NF-κB pathway, leading to aberrant expression. While we have exclusively investigated gene expression and not mutation status, exploring heterogeneity in the pan-cancer data rather than performing a global analysis might reveal further ROCK inhibitor vulnerabilities in these subtypes.

We have observed higher levels of Rho-ROCK-Myosin II signaling in AML cells, indicating that its activity could aid transformation, proliferation, survival or acquisition of stem-like properties. In addition, we have identified a subtype of AML, MLL-AFDN-r, that was highly responsive to ROCK inhibition compared to other karyotypes of unfavorable prognosis. In MLL-r cells, induction of leukemogenesis is mediated by KMT2A self-association and in cooperation with DOT1L increasing transcription of MLL-target genes.^93^^,^^94^ In MLL-AFDN cells, DOT1L inhibition led to cell differentiation and increased apoptosis.^95^ On the other hand, one of the mechanisms by which MLL-AFDN translocation induces leukemogenesis is via RAS activation^96^ in the cytoplasm and engagement of MAPK signaling. Afadin is a nectin- and actin-binding filament responsible for connecting nectin to the actin cytoskeleton^97^^,^^98^ and it is the only cytoplasmatic protein out of all the common MLL fusion partners.^99^ MLL-AFDN oncogenicity is linked to both increased transcriptional coactivation of KMT2A but also to delocalization of Afadin from the cytoplasm to the nucleus.^96^ Moreover, ROCK inhibitor treatment of some, but not all, AML cells resulted in reduced MAPK signaling,^32^ while in melanoma reduced BRAF-MAPK signaling led to reduced ROCK-Myosin II signaling.^12^ All these mechanisms could operate as possible drivers of increased sensitivity to ROCK inhibitors, and further work is guaranteed to expand these observations to other MAPK-driven malignancies. In addition, considering the success of ROCK2-specific inhibitors in the treatment of GvHD, it would be interesting to study the effect of these inhibitors in AML patients post-transplantation.

Our work suggests that the balance between differentiation and stemness could be predictive of ROCK inhibitor responses. From epithelial tumors to hematological malignancies, stem-like properties and de-differentiation are associated with ROCK inhibitor response. Moreover, within each tumor type, ROCK inhibition is more effective toward de-differentiated stem-like cells. ROCK inhibition has been reported to induce differentiation of cancer cells^23^^,^^84^^,^^100^ and its function in controlling stemness features has been defined in melanoma,^17^ breast cancer,^18^ and pancreatic cancer.^34^ Interestingly, ROCK inhibitor Y-27632 is used to culture organoids of epithelial cancers.^101^ This is due, in part, to the ability of Y27632 to moderately reduce contractility, thus allowing *in vitro* attachment.^102^^,^^103^^,^^104^^,^^105^ Other ROCK inhibitors may not have the same effect.^25^^,^^26^ Y-27632, a classic ROCK inhibitor with poor selectivity and potency is used at concentrations resulting in severe off-target effects,^25^^,^^28^ including higher inhibition potency against PKN and AGC kinases other than ROCK.^25^ We hypothesize that the addition of ROCK inhibitors to organoids would not have severe effects on the growth of the epithelial-like differentiated cancer cells, but could affect their secretory profile.^37^ Moreover, ROCK inhibitors impair the survival of cancer cells that have undergone EMT, which may be under-represented in cancer patient-derived organoid biology.

In the current work, we find that latest generation, potent and selective ROCK inhibitors either kill the cells or induce re-expression of melanoma differentiation markers within a 24-h treatment in the subset of cells that survive the treatment. AML MLL-r cells are in a highly de-differentiated state because MLL rearrangements activate genes involved in early development, promoting the expansion of immature progenitors.^65^^,^^66^^,^^93^^,^^99^ Importantly, Rho-ROCK signaling has been reported to promote drug resistance to targeted therapies in melanoma^106^ and to be a vulnerability of therapy-resistant cells.^12^ Moreover, in pancreatic cancer, ROCK inhibitors have been reported to sensitize pancreatic cancer stem cells to gemcitabine treatment^24^ and have also been proposed as a treatment in chemoresistant stem-like osteosarcoma cells,^23^ where ROCK inhibition induces terminal differentiation suppressing tumorigenesis. This body of work suggests that ROCK inhibitor treatment could be effective in targeting a subpopulation of cells with stemness, metastatic, and therapy-resistant properties.

Collectively, our data show that ROCK inhibition decreases cell viability in highly de-differentiated, stem-like and migratory cells and paves the way toward the use of ROCK inhibitors in the clinic. We propose a series of pan-cancer biomarkers that could guide further development of these drugs and the selection of appropriate patients in the clinic.

### Limitations of the study

While our pan-cancer analysis identifies ROCK dependency across multiple cancer types, several limitations should be considered. First, we used GDSC as a drug screening method for cell line interrogation, which employed different viability assays (Syto60 or Resazurin for GDSC1; CellTiter-Glo for GDSC2), potentially introducing assay-dependent variability in sensitivity measurements between adherent and suspension cells. Second, our study primarily relies on *in vitro* cell line models, which may not fully recapitulate the complexity of the tumor microenvironment and its influence on ROCK signaling. Third, while we show E-cadherin loss as a biomarker in epithelial cancers, the precise molecular mechanisms linking CDH1 loss to ROCK dependency warrant further investigation. Finally, our findings suggest an effect of ROCK inhibitors in organoid cultures; these conclusions are based on limited experimental conditions, and the optimal culture conditions that preserve clinically relevant cancer cell populations while maintaining organoid viability need to be systematically established.

## Resource availability

### Lead contact

Requests for further information and resources should be directed to and will be fulfilled by the lead contact, Victoria Sanz Moreno (victoria.sanz-moreno@icr.ac.uk).

### Materials availability

This study did not generate new unique reagents.

### Data and code availability

• This study analyzes existing, publicly available data. Gene expression datasets analyzed in this manuscript are available from NCBI GEO under the accession numbers GSE118963, GSE107490, GSE72061, GSE117994, GSE104406, GSE113182, GSE213119, and GSE23764. TCGA and TARGET cohorts are available from the National Cancer Institute GDC data portal. Cell lines microarray data are available in the Genomics of Drug Sensitivity in Cancer (GDSC) portal. BEAT-AML1.0-COHORT is available in the supplementary data of JW Tyner et al., Nature 2018 (30333627, Table S8). All data reported in this study will be shared by the lead contact upon request.
• This study does not report original code.
• Any additional information required to reanalyze the data reported in this study is available from the lead contact upon request.

## Acknowledgments

We thank Breast Cancer Now for funding this work as part of Program Funding to the Breast Cancer Now Toby Robins Research Center. Sanz Moreno lab was further supported by The Institute of Cancer Research, Cancer Research UK (CRUK) C33043/A24478; Barts Charity; Worldwide Cancer Research 22-0329 and UKRI grant reference EP/X033392.

## Author contributions

Conceptualization, V.S.-M. and J.B.; methodology, J.B., Y.T., O.M., J.A.J.M., S.G., J.S., A.P.-R., A.R.-M., K.K., J.F., and V.S.-M.; investigation, J.B., O.M., S.G., Y.T., J.A.J.M., D.D., A.P.-R., F.B.-C., W.W., L.N.v.d.L., C.J.L., and A.R.-M.; resources, A.C., J.G.G., T.H., I.M., J.F., and V.S.-M.; analysis, J.B., O.M., S.G., Y.T., J.A.J.M., F.B.-C., R.G., L.N.v.d.L., and C.J.L.; visualization, J.B. and V.S.-M.; manuscript writing, J.B. and V.S.-M.; funding acquisition, I.M., A.R.-M., K.K., J.F., and V.S.-M.; project administration, J.B. and V.S.-M.; supervision, V.S.-M.

## Declaration of interests

The authors declare no competing interests.

## STAR★Methods

### Key resources table

**Table:** 

REAGENT or RESOURCE	SOURCE	IDENTIFIER
**Antibodies**		

CD44	Cell Signaling Technology	Cat#: 3570; RRID:AB_2076465
E-cadherin	Cell Signaling Technology	Cat#: 3195; RRID:AB_2291471
GAPDH	Milipore	Cat#: MAB374; RRID:AB_2107445
HMB45	Abcam	Cat#: Ab787; RRID:AB_306146
MLC2	Cell Signaling Technology	Cat#: 3672; RRID:AB_10692513
p65 (C22B4)	Cell Signaling Technology	Cat#: 4764; RRID:AB_823578
pMLC2 (ser19)	Cell Signaling Technology	Cat#: 3671; RRID:AB_330248
pMLC2 (Thr18/Ser19)	Cell Signaling Technology	Cat#: 3674; RRID:AB_2147464
p-p65 (Ser536)	Cell Signaling Technology	Cat#: 3033; RRID:AB_331284
RhoA (67B9)	Cell Signaling Technology	Cat#: 2117; RRID:AB_10693922
SOX10	Abcam	Cat#: ab155279; RRID:AB_2650603
GAPDH	Abcam	Cat#: Ab8245; RRID:AB_2107448
Sheep anti-Mouse IgG, HRP-linked whole Ab	Cytiva	Cat#: NA931; RRID:AB_772210
Donkey anti-Rabbit IgG, HRP-linked whole Ab	Cytiva	Cat# NA934; RRID:AB_772206
Goat anti-Mouse IgG (H + L) Cross-Adsorbed Secondary Antibody, Alexa Fluor 488	Thermo Fisher Scientific	Cat# A11008; RRID:AB_2534069
Goat anti-Mouse IgG (H + L) Cross-Adsorbed Secondary Antibody, Alexa Fluor 647	Thermo Fisher Scientific	Cat# A21235; RRID:AB_2535804
Goat anti-Rabbit IgG (H + L) Cross-Adsorbed Secondary Antibody, Alexa Fluor 488	Thermo Fisher Scientific	Cat# A11009; RRID:AB_2534076
Goat anti-Rabbit IgG (H + L) Cross-Adsorbed Secondary Antibody, Alexa Fluor 647	Thermo Fisher Scientific	Cat# A21244; RRID:AB_2535812

**Biological samples**		

Human blood malignancies tissue microarray	Barts Cancer Institute, QMUL	NA

rowhead**Chemicals, peptides, and recombinant proteins**		

GSK269962	Axon MedChem	Cat#: 1167; CAS: 850664-21-0
H1152	Calbiochem	Cat#: 555550; CAS: 871543-07-6
LY2409881	MedChemExpress	Cat#: HY-B0788A; CAS: 946518-60-1
Atelopeptide Collagen I	Advanced Biomatrix	Cat#: 5005
MethoCult H4434 Classic	StemCell Technologies	Cat#: 04434
Phalloidin Alexa Fluor 546	Thermo Fisher Scientific	Cat#: A-22283
Phalloidin Alexa Fluor 647	Thermo Fisher Scientific	Cat#: A-22287

**Experimental models: Cell lines**		

A375P	Prof. Richard Hynes (HHMI, MIT, USA)	Clark et al.^53^
A375M2	Prof. Richard Hynes (HHMI, MIT, USA)	Clark et al.^53^
A375P TB	Prof. Victoria Sanz-Moreno (ICR, UK)	Georgouli et al.^13^
A375P IF	Prof. Victoria Sanz-Moreno (ICR, UK)	Georgouli et al.^13^
4T1	Prof. Gilbert Fruhwirth (KCL, UK)	Lelekakis et al.^50^
4T1.2	Prof. Gilbert Fruhwirth (KCL, UK)	Lelekakis et al.^50^
T47D Cas9	Prof. Chris Lord (ICR, UK)/This study	NA
T47D CDH1^−/−^	Prof. Chris Lord (ICR, UK)/This study	NA
MCF7 Cas9	Prof. Chris Lord (ICR, UK)	Bajrami et al.^49^
MCF7 CDH1^−/−^	Prof. Chris Lord (ICR, UK)	Bajrami et al.^49^
MDA-MB-231-TGL	Prof. Joan Massagué (MSK, USA)	Bos et al.^51^
MDA-MB-231-BrM2-TGL	Prof. Joan Massagué (MSK, USA)	Bos et al.^51^
NB4	Prof. Jude Fitzgibbon (BCI, UK)	NA
ME-1	Prof. Jude Fitzgibbon (BCI, UK)	NA
MOLM-13	Prof. Jude Fitzgibbon (BCI, UK)	NA
THP-1	Prof. Jude Fitzgibbon (BCI, UK)	NA
MV4-11	Prof. Jude Fitzgibbon (BCI, UK)	NA
ML-2	DSMZ	ACC 15
SHI-1	DSMZ	ACC 645

**Oligonucleotides**		

Human Non-targeting ON-TARGET plus siRNA UGGUUUACAUGUCGACUAA	Dharmacon	Cat#: D-001810-01-20
Human RELA ON-TARGET plus siRNA CCCACGAGCUUGUAGGAAA	Dharmacon	Cat#: J-003533-07-0005
Human non-targeting siRNA siGENOME UGGUUUACAUGUCGACUAA	Dharmacon	Cat#: D-001210-05-05
Human IL1R1 siGENOME SMARTpool GAACAAGCCUCCAGGAUUC; GGACUUGUGUGCCCUUAUA; GAACACAAAGGCACUAUAA; CAAUUGAUCUGUAAUGUCA	Dharmacon	Cat#: M-005188-00-0005
Human IL1A siGENOME SMARTpool GGAUGAAGCAGUGAAAUUU; GAUCAUCUGUCUCUGAAUC; GAAAUCCUUCUAUCAUGUA; GGCCAAAGUUCCAGACAUG	Dharmacon	Cat#: M-007952-01-0005
Human IL1R1 QuantiTect Primer Assay	Qiagen	Cat#: QT00081263
Human IL1A QuantiTect Primer Assay	Qiagen	Cat#: QT00001127
Human GAPDH QuantiTect Primer Assay	Qiagen	Cat#: QT00079247

**Software and algorithms**		

RStudio	https://posit.co/downloads/	RRID:SCR_000432
FIJI (ImageJ)	http://fiji.sc	RRID:SCR_002285
QuPath	https://qupath.github.io/	RRID:SCR_018257
ZEISS ZEN	https://www.zeiss.com/microscopy/us/products/software/light-microscopy-software.html	RRID:SCR_013672
Gene Pattern	http://www.broadinstitute.org/cancer/software/genepattern/	RRID:SCR_003201
Morpheus	https://software.broadinstitute.org/morpheus/	RRID:SCR_017386
Graphpad PRISM 10	https://www.graphpad.com/features	RRID:SCR_002798
Sartorius IncuCyte S3 Live Cell Analysis System	https://www.sartorius.com/en/products/live-cell-imaging-analysis/live-cell-analysis-software	RRID:SCR_023147

### Experimental model and study participant details

#### Cell lines

Human melanoma cell lines A375P and A375M2 (human, melanoma, female) were obtained from Prof. Richard Hynes (HHMI, MIT, USA). Human breast cancer cell lines MDA-MB-231-TGL and MDA-MB-231-BrM2-TGL (abbreviated as 231 and 231-BrM2; human, breast adenocarcinoma, female) were obtained from Prof. Joan Massagué (MSK, USA). Human AML cell lines NB4 (female), ME-1 (male), THP-1 (male), MOLM-13 (male), and MV4-11 (male) were obtained from Prof. Jude Fitzgibbon (BCI, QMUL, UK). Human AML cell lines ML-2 (male) and SHI-1 (male) were purchased from DSMZ (Leibniz Institute, Germany). MCF-7 and T47D (wild-type and CDH1^−/−^) human female breast cancer cell lines were obtained from Prof. Christopher J. Lord (ICR, UK). Mouse female breast cancer cell lines 4T1 and 4T1.2 were obtained from Prof. Gilbert Fruhwirth (KCL, UK). All cell lines were maintained in DMEM, RPMI-1640 or IMDM medium supplemented with 5–10% FBS and 1% penicillin/streptomycin at 37°C in a humidified atmosphere with 5% CO_2_ or 10% CO_2_ for melanoma. Cell line authentication was performed by short tandem repeat (STR) profiling. Mycoplasma testing was conducted weekly when cells were in culture.

#### Animal studies

Tumor sections from *in vivo* studies of 4T1 breast cancer cell-derived tumors and A375P and A375M2 melanoma cell-derived tumors have been obtained from previously published studies.^17^^,^^37^^,^^59^

A375M2 pre-treatment *in vivo* study was performed by pre-treating A375M2 cells with 5 μM of H1152 for 5 days changing media daily. 1x10^6^ cells were mixed in 100 μL of Growth Factor Reduced Matrigel (Corning) and injected subcutaneously into female NOD/SCID/IL2Rγ− (NSG) mice. At the end of the experiment, tumors were formalin-fixed and paraffin-embedded (FFPE).^17^ Mice were housed under specific pathogen-free conditions in groups of 4–5 animals per cage with food and water provided *ad libitum*. Environmental conditions were maintained at 21 ± 1 °C with 40–60% relative humidity under a 12-h light/dark cycle. All mice were purchased from Charles River UK and were 6–12 weeks of age at the time of experiments. Animal procedures were conducted in accordance with UK Home Office regulations under The Animals (Scientific Procedures) Act 1986. Ethical approval was obtained from the Ethical Review Process Committee at The Francis Crick Institute, with experiments performed under UK Home Office project licenses (PPL P83B37B3C license).

A375P and A375M2 *in vivo* study was performed in NXG (NOD-*Prkdc*^*scid*^*-Il2rg*^*tm1*^*/Rj*) female mice 6–8 weeks old obtained from Janvier Labs. Mice were kept at the QMUL Biological Services holding facility and maintained at 7 h light/dark cycle, ambient temperature of 19–22°C and humidity of 50–60%. 2x10^5^ A375P and A375M2 cells in a volume of 50 μL in PBS^−/−^ were intradermally injected into the flank of NXG mice, with two injections per mouse, producing tumors in two flanks (*n* = 8 mice and *n* = 16 tumors per condition). For A375M2-derived tumors, only one timepoint was considered (24 days; *n* = 16 tumors) while for A375P two different timepoints were considered (24 days, *n* = 8 tumors; 36 days, *n* = 8 tumors).^59^ Ethical approval for animal studies was obtained from the Ethical Review Process Committee at Barts Cancer Institute, and all procedures were performed under UK Home Office project licenses (PPL PP4475211 license).

4T1 murine breast cancer cells resuspended in Matrigel were orthotopically injected in the mammary fat pad of Balb/CJ background female mice and tumors were harvested after 14 days.^37^ Animals were handled according to the Institutional Committees on Animal Welfare of the UK Home Office and under specific pathogen-free conditions. Ethical approval was obtained from the Ethical Review Process Committee at The Francis Crick Institute, with experiments performed under UK Home Office project licenses (PPL P83B37B3C license).

#### Patient samples

Primary AML samples were obtained from bone marrow biopsies and isolated at the Barts Cancer Institute (BCI) or Finnish Institute for Molecular Medicine (FIMM) tissue bank facilities, where they were stored in liquid nitrogen. All patients provided informed consent for the storage and use of their samples for research purposes. Sample collection and experimental procedures were performed in accordance with approvals from the Local Research Ethics Committee (London - City and East, REC ref. 07/Q0603/5) and in accordance with the Helsinki declaration. RNA-seq data and drug sensitivity profiles for these samples were obtained from NCBI Gene Expression Omnibus (GEO) under accession number GSE213119.

Tissue microarrays (TMA) contain formalin-fixed paraffin-embedded (FFPE) human samples containing different blood malignancies were included in the case series. Each patient was represented by three cores (1 mm diameter). Samples were obtained from the center for Haemato-oncology at the Barts Cancer Institute. Samples were collected with specific informed consent, in accordance with the Helsinki Declaration. Local ethical approval was obtained for this work, under the London Research Ethics Committee of the East London and the City Health authority (REC ref. 10/H0704/65 and 05/QO605/140).

### Method details

#### Cell culture on collagen I matrices

Cells were plated on top of thick collagen matrices as previously described in^17^ using atelopeptide Collagen I (#5005, Advanced Biomatrix) at a final concentration of 1.7 mg/mL and left to polymerize for 4 h. Cells were seeded on top of the collagen matrix in DMEM medium 10% FBS and left overnight. Media was changed to DMEM 1% FBS ± DMSO/inhibitors at different timepoints and fixed in 4% paraformaldehyde prior staining and imaging.

#### Drug treatments

The following drugs were used in the study at the indicated concentrations: GSK269962 (0.1 μM, 0.5 μM, 1.5 μM or 5 μM in DMSO; Axon Medchem), H1152 (5 μM in water; Calbiochem) dissolved in 1% FBS supplemented DMEM or RPMI media. For each control, the same volume of DMSO as the highest drug concentration used was employed.

#### Pulldown assays

300.000 cells were seeded in 6 cm petri dishes and left 24 h in DMEM medium supplemented with 10% FBS. After 24 h, medium was changed to 1% FBS and treatment was added as indicated. 24 h after seeding, cells were washed once with ice-cold PBS and lysed in 350 μL of pulldown lysis buffer (1x TBS, 10 mM MgCl2, 1 mM DTT, 0.1 mM PMSF + protease inhibitor tablet). Samples were sonicated and centrifuged for western blot. 30 μL of the sample was separated in order to measure total protein by western blot, while 280 μL of the sample were combined to 18 μL of Rhotekin-RBD Beads (Cytoskeleton) and incubated at 4°C with agitation for the pulldown. After incubation, the pulldown fraction was washed with pulldown lysis buffer and centrifuged at 700g for 4 min to pellet the beads twice and the beads were resuspended in 30 μL of Laemmli lysis buffer.

#### siRNA transfections

250.000 cells were seeded in 6-well plates in a total volume of 2.5 mL combined with 0.5 mL of transfection mix containing 7.5 μL of Lipofectamine 2000 (Invitrogen), 500 μL of Optimem 1X (GIBCO) and 20 nM siGenome Smartpool or individual OTs (On-target) siRNA oligonucleotides (Dharmacon). 24 h after transfection, medium was changed to DMEM 10% FBS or reseeded on top of collagen plates. 48 h after transfection, medium was changed to DMEM 1% FBS and seeded for downstream experiments. Oligonucleotide sequences as follow (key resources table).

#### Survival on collagen

5000 cells were added on top of a thick collagen matrix in DMEM 10% FBS. After 24 h, medium was changed to DMEM 1% FBS with the drug or the same volume of DMSO as drug used. Cells were left in the incubator for 72 h before fixation in 4% PFA/PBS. After fixation, cells were washed in PBS with Hoechst for nuclear staining. Wells were imaged in the Revolve fluorescent microscope (Echo) at 4X and analyzed with FIJI (ImageJ) applying watershed and counting objects filtering for size >50. Cell growth on collagen was assessed as number of cells (object count from FIJI) in metastatic cell line divided by number of cells in parental cell lines after 72 h in DMSO conditions.

#### CRISPR/Cas9 targeting and E-cadherin isogenic cell lines

MCF7 CDH1 wild type and CDH1^−/−^ cells are described in.^49^ Similar T47D CDH1 wild type and CDH1^−/−^ cells were generated as in^49^; *CDH1* in wild type cells was targeted using Edit-R-CRISPR-CAS9 gene engineering kit (GE Dharmacon) with the following crRNA sequence: 5′-GCUGAGGAUGGUGUAAGCGAGUUUUAGAGCUAUGCUGUUUUG-3′. E-cadherin knockout was confirmed by western blotting.

#### Tumor cell isolation from tumor body and invasive front

Cells from the tumor body and invasive front of A375P-derived tumors were isolated as described in^13^. Tumors were surgically removed from mice and immediately placed into falcon tubes containing PBS without CaCl_2_/MgCl_2_, then maintained on ice until further processing. Each tumor was bisected longitudinally using a scalpel; one-half was fixed in 4% formaldehyde for 48 h prior to paraffin embedding, and the remaining half was processed for cell isolation. For cell isolation, the tumor half was carefully dissected using forceps, scissors, and a scalpel to separate two morphologically distinct regions: the peritumoral area, identified by its translucent appearance, and the tumor core, which presented as a dense, darker tissue. Both regions were cut into small fragments and subjected to enzymatic digestion in 1 mL of digestion buffer per sample at 37°C with agitation. The digestion buffer consisted of 90 μL Liberase TM (5 mg/mL; Roche), 90 μL Liberase TH (5 mg/mL; Roche), 30 μL DNase I (5 mg/mL; Sigma), and 6 mL HBSS (GIBCO).

#### Phase contrast brightfield microscopy

For cell morphology quantification, a Zeiss Axiocan 202 mono camera was used, and images were processed using Zeiss Zen Blue software. Five representative images of cells in collagen were taken at 20X magnification including 30–60 cells per picture, quantifying a total of 100–150 cells per experiment. To quantify morphology, FIJI (ImageJ) was used a software by circling the shape of the individual cell using the “Freehand selection tool” and measuring the “roundness index” included in the “shape descriptors” option within FIJI.

#### Antibodies

Antibodies used for western blotting, immunofluorescence and immunohistochemistry listed in key resources table. For Western Blot, the following dilutions were used for each antibody: CD44 (1/2000), E-cadherin (1/1000), GAPDH (1/10000), MLC2 (1/1000), p65 (1/1000), pMLC2 thr18/Ser19 (1/1000), p-p65 Ser536 (1/1000), Rhoa (1/750), SOX10 (1/2000). For immunofluorescence, the following dilutions were used for each antibody: p65 (1/200), pMLC2 Ser19 (1/200). For immunohistochemistry, the following dilutions were used for each antibody: HMB45 (1/300), p65 (1/100), pMLC2 Ser19 (1/200), SOX10 (1/250).

#### Microscopy

Immunofluorescence was performed on cells seeded on top of coverslips (2D) or seeded on top of a thick collagen I matrix (2.5D). Cells were fixed in 4% PFA/PBS for 15 min and permeabilized in 0.3% Triton X-100 in 4% BSA/PBS, followed by overnight staining. In nuclear/cytoplasmatic experiments, cells were permeabilized with 0.5% Triton and 0.2% Triton was also added to the primary incubation. Cells were then incubated with their corresponding fluorophore-conjugated secondary antibody (Thermo Fisher Scientific) at a 1/1000 dilution and Alexa Fluor-conjugated Phalloidin antibody for labeling F-actin at a 1/400 dilution. Before imaging, cells were stained with Hoechst 33342 (5 μg/mL) at 1/2000 dilution for nuclear staining. Coverslips were then mounted on top of SuperFrost Plus slides using Fluoroshield mounting media and imaged on Zeiss LSM710 confocal microscope, and processed in the Zeiss Zen software, or Super Resolution Spinning Disk (TIRF/STORM/SoRa) equipped with a 63×/1.4 NA oil-immersion objective. Images were analyzed using FIJI (ImageJ).

#### Colony forming units (CFU)

CFU were carried out using MethoCultTM H4434 (StemCell Technologies) methylcellulose semisolid media supplemented with 1% penicillin/streptomycin. Cells were resuspended in Iscove’s MDM (IMDM) media supplemented with 2% FBS and mixed with methylcellulose media prior seeding. After 7–14 days, depending on the cell line, colonies were quantified and measured using Incucyte (Sartorius) and Incucyte analysis software.

#### RT-qPCR

QuantiTect Primer Assays (Qiagen) and Brilliant III SYBR Green RT-qPCR 1-step system (Agilent Technologies) were used following the manufacturer’s instructions. Between 50 and 100 μg of RNA were used per reaction, performed in 3 technical replicates per condition and using GAPDH as a control. RT-qPCR experiments were performed on an ABI QuantStudio 7 PCR/OneStep plus machine and later analyzed using Real-Time PCR/OneStep Plus software. Primers sequences shown in key resources table.

#### Western blotting

Samples for western blotting were lysed in Laemmli lysis buffer, boiled for 5 min and sonicated using a VCX Ultrasonic processor in cold water (4°C) for 2 cycles on/off of 15 s at 75% amplitude to ensure adequate MLC2 extraction. Samples were then centrifuged prior addition of 4X LDS sample buffer at 13.000 rpm for 30 min and loaded into 12% NuPage Tris-Glycine gels using either 1X Novex MES-SDS or 1X Novex MOPS-SDS Running buffers followed by transfer into PDVF membranes (0.45 μm, Immobilion). Membranes were then incubated at 4°C overnight with primary antibodies at the described concentration. Membranes were then developed incubated with HRP-conjugated secondary antibodies ECL Rabbit IgG, HRP-linked whole Ab and ECL Mouse IgG, HRP-linked whole Ab (Amersham) at a 1/10.000 dilution and detected using ECL Plus or Forte detection system (GE Healthcare) for 2 min and imaged on an Amersham Imager 600 (GE Healthcare).

#### Gene set enrichment analysis (GSEA)

GSEA was performed and visualized using the Clusterprofiler package^107^ in RStudio. Enrichment was performed using the Hallmarks, Reactome and Gene Ontology databases from Molecular Signature Database (MSigDb). Single sample GSEA (ssGSEA) was performed in GenePattern (Broad Institute) and normalized the values as *Z* score.

#### Genomics of Drug Sensitivity in Cancer (GDSC)

GDSC database^41^ was used to obtain baseline transcriptomic data of pan-cancer cell lines as well as sensitivity profiles to ROCK inhibitors GSK269962, GSK429286 and Y-39983 from GDSC2. Cell lines were either sourced from commercial vendors or academic collaborators, grown in DMEM or RPMI supplemented with 5% or 10% FBS and penicillin/streptomycin and maintained at 37 °C and 5% CO^2^. Sensitivity profiles were obtained from the measurement AUC (area under the curve) and IC_50_. Drug sensitivity data from GDSC represent cell viability measurements after 72-h drug exposure, determined by ATP quantification using CellTiter-Glo.

Baseline transcriptomic data was obtained from 100 cell lines representing 30 different types of solid cancers and separated according to IC_50_. For pan-cancer solid comparison, the following cell lines were used where responder cell lines showed an IC_50_ < 0.61 μM and non-responders IC_50_ > 68.5 μM to GSK269962. Responders: BB49-HNC, U031, D-566MG, TC-YIK, Hs-633T, IOSE-523, HSC-3, RCC-JF, GCT, SW872, GI-ME-N, UMC-11, A375, SW1088, NTERA-2-cL-D1, EFO-27, D-336MG, KELLY, KS-1, IMR-5, NCI-H292, OV-7, SBC-1, SW48, SK-LMS-1, CAL-62, MSTO-211H, NCI-H810, Hs-940-T, MES-SA, SK-CO-1, CAS-1, SW982, BT-549, SK-HEP-1, YH-13, IOSE-397, HOP-92, HN, C-33-A, HT-1080, HCC-44, SNU-407, LK-2, D-263MG, HOS, KYSE-70, NCI-H1975, FU97, NCI-H847. Non responders: CHP-126, NCI-H1092, MZ7-MEL, LB2518-MEL, NCI-H1648, COLO-783, HCC2157, SK-MEL-30, NCI-H2804, LB373-MEL-D, DMS-53, NCIH20877, KURAMOCHI, KM12, KYSE-140, COLO-680N, LC-1-sq, HCC1419, OAW-28, HCC1143, SU8686, NCI-H1666, CAPAN-1, KNS-62, SK-MEL-3, PANC-08-13, SNU-61, OV-17R, CAL-85-1, NCI-N87, Hs-766T BT-20, SW837, CAPAN-2, NCI-H1623, NCI-H2444, EGI-1, NCI-H513, KYSE-520, COLO-829, COLO-678, UWB1.289, SW948, BT-483, OVKATE, CL-40, NCI-H2066, PANC-04-03, NCI-H2291. For melanoma, 17 cell lines were selected according to sensitivity to GSK269962, where responders showed an IC_50_ < 2 μM and non-responders IC_50_ > 50 μM.

#### RNA-seq and sensitivity profile of AML patient samples

RNA-seq data of AML patients is available from GEO under accession number GSE213119. Patient samples were separated according to their drug sensitivity score (sDSS) to GSK269962 into responders (sDSS >2 for karyotype separation and sDSS >5 for RNA-seq analysis) and non-responders (sDSS <0). For drug sensitivity experiments of primary AML samples, the chemical compounds, DMSO (negative control) and benzethonium chloride (positive controls) were added to 384-well plates using an acoustic liquid dispensing system ECHO 500/550 (Labcyte). Frozen samples from peripheral blood or bone marrow were thawed and resuspended in conditioned media (77.5% RPMI 1640, 10% FCS, 12.5% human HS-5 bone marrow stromal cell line conditioned media and 1% penicillin/streptomycin) followed by cell recovery and live cell count. Drugs were first dissolved in cell-free medium followed by 4X volume of cell suspension containing 5000 live cells using EL 406-plate washer-dispenser (BioTek). Cells were exposed to 10-fold concentrations of each compound for 72 h and viability was assessed using Cell-Titer Glo (CTG) protocol and the PHERAstar FS multimode plate reader (BMG Labtech). AUC of the drug response curves was used in order to calculate the selective drug sensitivity score (sDSS) comparing the effects in leukemic cells taking into consideration two healthy donor samples included for screening.

#### AML MLL expression analysis

Normalized FPKM or RPKM expression data were sourced as follows: A) TCGA-LAML data was downloaded as RPKM from the National Cancer Institute GDC data portal. B) BEATAML1.0-COHORT data was downloaded as log RPKM values from supp data of JW Tyner et al., Nature 2018.^108^ C) TARGET-AML data was downloaded as RPKM from the National Cancer Institute GDC data portal (dbGaP Accession: phs000465.v23.p8). D) FPKM and RPKM values for control (healthy HSCs and progenitor cells) and AML datasets came from NCBI GEO datasets GSE118963, GSE107490, GSE72061, GSE117994, GSE104406, and GSE113182. Datasets were batch-corrected and combined using the mnnCorrect function from the batchelor package in R.

#### AML karyotype confirmation cohort

Retrospective samples from BM and PB, at diagnosis or treatment naive, were collected from all cases and total RNA extracted using the MagNA Pure 96 Instrument and the MagNA Pure 96 Cellular RNA LV Kit (Roche LifeScience, Mannheim, Germany). 250 ng of RNA were used as input for the TruSeq Total Stranded RNA kit with rRNA depletion (Illumina, San Diego, CA, USA). Libraries were sequenced on the NovaSeq6000 with a median of 50 million reads per sample. Reads were mapped with STAR aligner (v2.5.0a) to the human reference genome hg19 (RefSeq annotation). Gene and transcript specific read abundance was calculated with Cufflinks (v2.2.1). For gene expression analysis, estimated read counts for each gene were normalized by applying Trimmed mean of M-values (TMM) normalization method and the resulting log2 counts per million (CPMs) were used. The enrichment of the ROCK signature in the individual samples was calculated with ssGSEA 2.0 (https://github.com/broadinstitute/ssGSEA2.0).

#### Immunohistochemistry

Tumors were fixed in formalin and embedded in paraffin prior section cuts of 4 mm thickness. Sections were incubated for 20 min at 60°C, followed by rehydration and blocking of endogenous peroxidase activity with 2% hydrogen peroxidase for 10 min. For antigen retrieval, citric-based (pH = 6) buffer (Vector labs) was used for 7 min at 110°C. Samples were then circled with a hydrophobic PAP pen before blocking for 30 min with 1% BSA/PBS and incubated overnight at 4°C with the corresponding primary antibody diluted in Emerald antibody diluent (Sigma). The following day, samples were washed with 1X DAKO wash buffer prior to incubation with biotinylated secondary antibodies (Vector labs) for 40 min at room temperature. Samples were developed using VIP substrate kit (Vector labs) for 10 min at room temperature, counterstained with Haematoxylin, dehydrated and mounted using DPX mounting media (Sigma).

Qupath^109^ was used for IHC quantification. H-score was used to represent intensity of the staining. H-score is calculated by separating individual cells by expression of the protein in 4 different categories: 0 (no staining), 1 (weak), 2 (intermediate) and 3 (high). H-score was then calculated as follows:

H−score=(%cells1+)×1+(%cells2+)×2+(%cells3+)×3

#### Schematics and illustrations

Schematics and illustrative figures presented in this study were created using BioRender (www.BioRender.com). All figures were exported and published in accordance with BioRender’s academic publication guidelines and licensing agreements.

### Quantification and statistical analysis

Statistical analysis of experimental data was performed using Graphpad PRISM 10. Unpaired *t* test and Mann-Whitney tests were used when comparing only two conditions with Gaussian and non-Gaussian distributions, respectively. When comparing a population to a set mean (1 in the case of fold change or 100 when measuring activity), sample *t* test was used to the number used in the control population. One-way ANOVA tests were used for multiple comparisons in which the values of the control population are different (non-control normalized prior statistical tests), either by comparing the mean of each population against the mean of every other population or by comparing the mean of each population against the mean of the control population (Tukey multiple comparisons test). For survival analysis, Kaplan-Meier curves show log rank statistical test or Gehan-Breslow-Wilcoxon test for early timepoints, performed in R using the Survival package. GSEA statistical values were also obtained in R using the ClusterProfiler package. For all statistical analyses: ns = not significant, ∗*p* < 0.05, ∗∗*p* < 0.01, ∗∗∗*p* < 0.001, ∗∗∗∗*p* < 0.0001.

### Additional resources

#### Clinical trials

Phase I/II open label study of belumosudil mesylate alone, and in combination with dexamethasone, in patients with relapsed/refractory multiple myeloma; ClinicalTrials.gov Identifier: NCT06105554; https://clinicaltrials.gov/study/NCT06105554.
