# Supplementary material for: Pan-cancer analysis reveals Rho kinase addiction as a vulnerability of de-differentiated cancer cells
Source: iScience. 2026 Jun 2;29(6):116031. doi: 10.1016/j.isci.2026.116031 (PMC13309995; doi:10.1016/j.isci.2026.116031)

iScience, Volume *29*

Supplemental information

Pan-cancer analysis reveals Rho kinase addiction as a vulnerability of de-differentiated cancer cells

Jaume Barcelo, Yumiko Teigen, Joshua Alexander James Martin, Samantha George, Dipanwita Das, Joanne Sewell, Anna Perdrix-Rosell, Findlay Bewicke-Copley, Ritobrata Ghose, William Yang, Rachel Brough, Andrew Clear, Wencke Walter, Torsten Haferlach, Ilaria Malanchi, John G. Gribben, Louie N. van de Lagemaat, Kamil R. Kranc, Christopher J. Lord, Ana Rio-Machin, Oscar Maiques, Jude Fitzgibbon, and Victoria Sanz-Moreno

This file includes:

Figures. S1 to S6
Figure legends. S1 to S6
Raw microscopy images for immunofluorescence experiments

Data S1_Figures S1 to S6 and figure legends S1 to S6

SUPP FIG 1

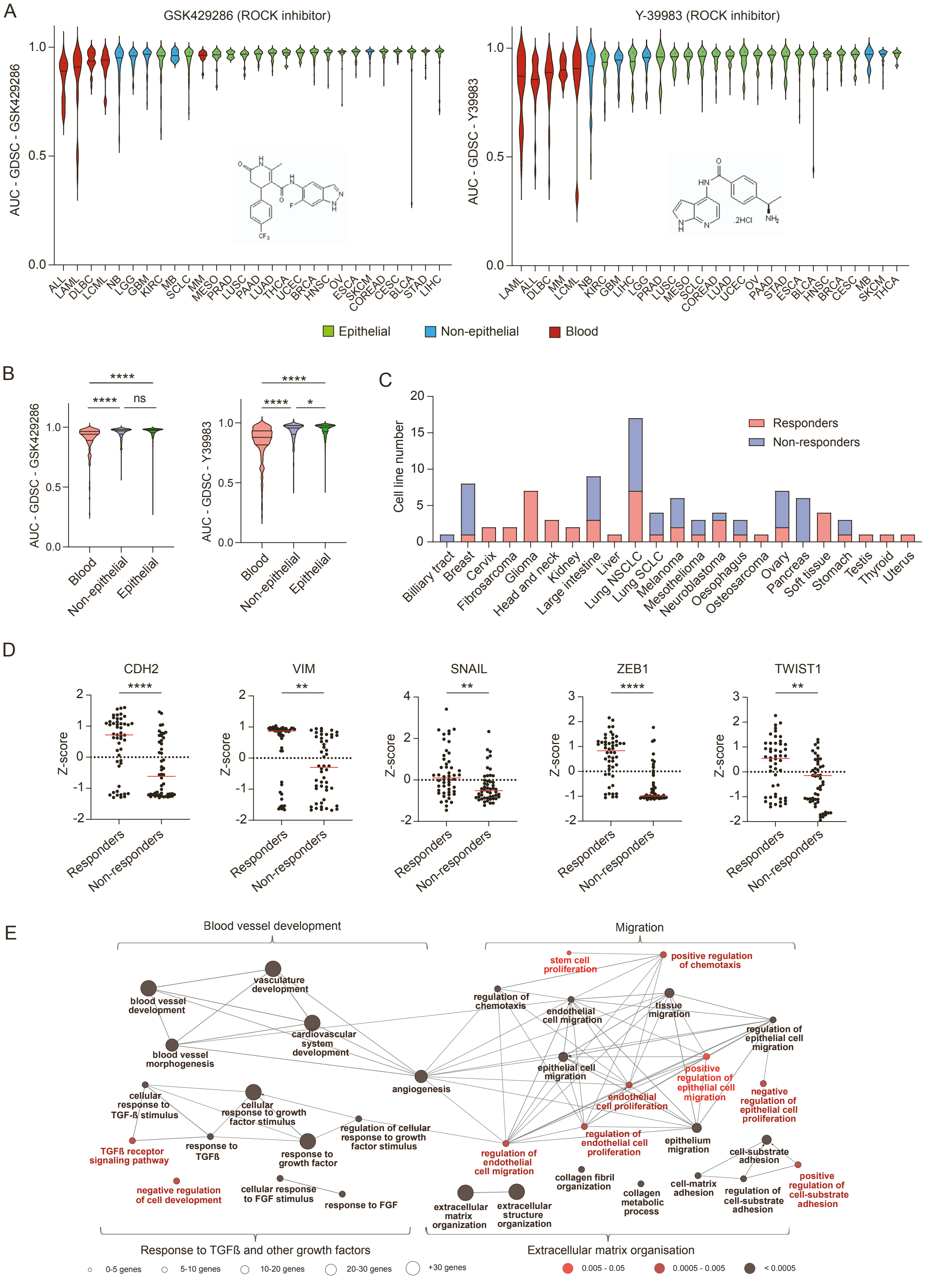

Fig. S1. ROCK inhibitor-responder tumor cells are in a unique transcriptional program

(A) Violin plot of pan-cancer distribution of GSK429286 and Y-39983 cell line sensitivity ranked from lowest to highest AUC response. Red: hematological malignancy; blue: solid cancer of non-epithelial origin; green: solid tumour of epithelial origin.

(B) AUC drug response across all cell lines separated by three categories according to their tumor of origin from (A). Statistical test: multiple comparisons by one-way ANOVA. Violin plot with interquartile range and min to max values. For GSK429286: n=148 cell lines (hematological); n=212 cell lines (non-epithelial) and n=482 cell lines (epithelial). For Y-39983: n=145 cell lines (hematological); n=194 cell lines (non-epithelial) and n=507 cell lines (epithelial).

(C) Number of cell lines included in the 100-cell line selection from GDSC for differential gene expression by tumor type. n=50 cell lines (responders) and n=50 cell lines (non-responders).

(D) Comparison of mRNA expression from figure 1E; dot plots show all points; red line shows median value. Statistical test: unpaired t test. n=50 cell lines (responders) and n=50 cell lines (non-responders).

(E) Pathway Enrichment Analysis (PEA) visualization with ClueGO of genes upregulated in ROCK inhibitor responder cell lines. Gene threshold: DGE > 1.3; p value < 0.05. Gene Ontology Biological Processes (GOBP) used for term enrichment.

For (B), (D): ns = not significant, * $p < 0.05$, ** $p < 0.01$, *** $p < 0.001$, **** $p < 0.0001$.

SUPP FIG 2

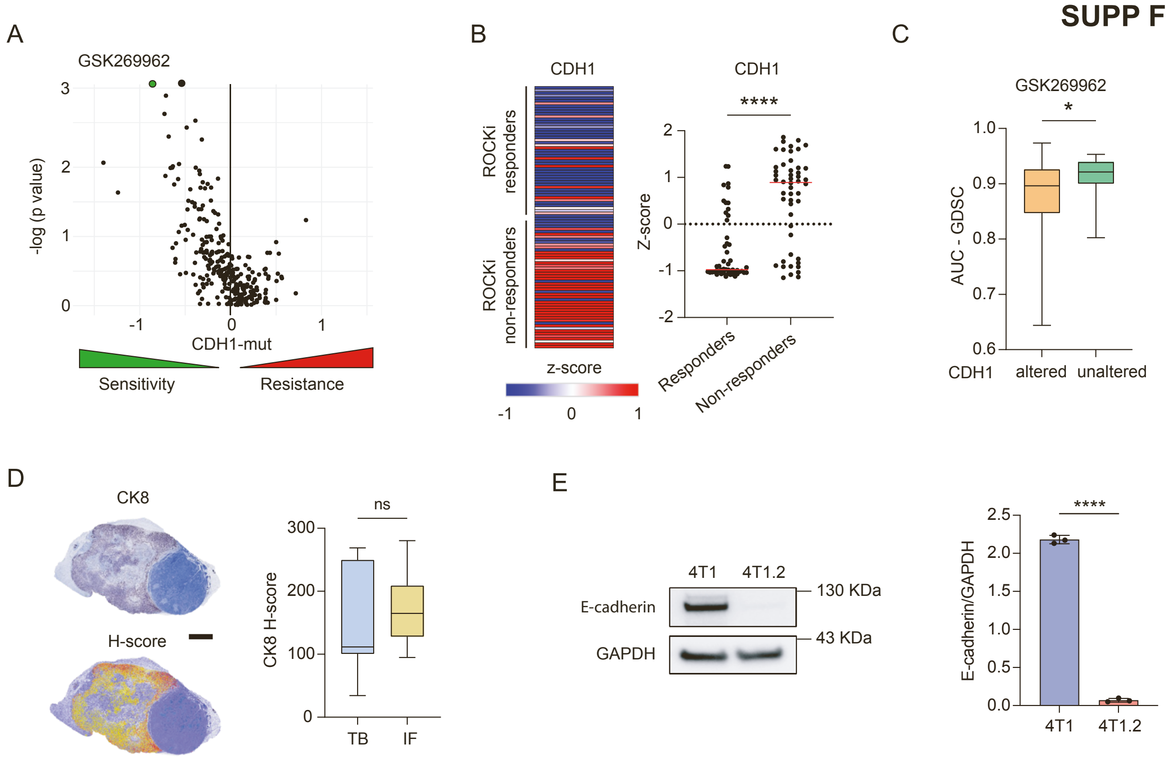

FIG S2: **E-cadherin loss is associated with ROCK inhibitor response in epithelial cancer cells**

(A) Dot plot showing differential sensitivity to GDSC inhibitors in CDH1-mut cell lines pan-cancer. Negative values correlate with higher sensitivity in mutant cell lines.

(B) mRNA expression heatmap of *CDH1* across the 100 cell lines included in the transcriptomic study at pan-cancer level comparing ROCK inhibitor responder and ROCK inhibitor non-responder cell lines (left) and z-score value quantification (right). All points shown, red line indicates median value. Statistical test: Unpaired t-test. n=50 cell lines (responders) and n=50 cell lines (non-responders).

(C) AUC sensitivity values of all BRCA cell lines included in DepMap separated into CDH-1 functional and CDH-1 altered groups. Box shows median value with interquartile range with whiskers showing min to max values. Statistical test: unpaired t test. n=13 cell lines (CDH1-altered) and n=31 cell lines (CDH1 unaltered).

(D) Representative images from CK8 staining in 4T1-derived tumors with H-score Qupath map (left) and quantification of staining in H-score in tumor body and invasive front of the tumors (right). Box shows median value with interquartile range with whiskers showing min to max values. Statistical test: unpaired t test. Scale bar: 1 mm. n=13 tumors; TB and IF quantified within each tumor.

(E) Western blot of E-cadherin and GAPDH in 4T1 and 4T1.2 cells (left) and quantification (right). Bar plot shows mean value with whiskers showing SD. Statistical test: unpaired t test. n=3 biological replicates.

For (B), (C), (D), (E): ns = not significant, * $p < 0.05$, ** $p < 0.01$, *** $p < 0.001$, **** $p < 0.0001$.

SUPP FIG 3

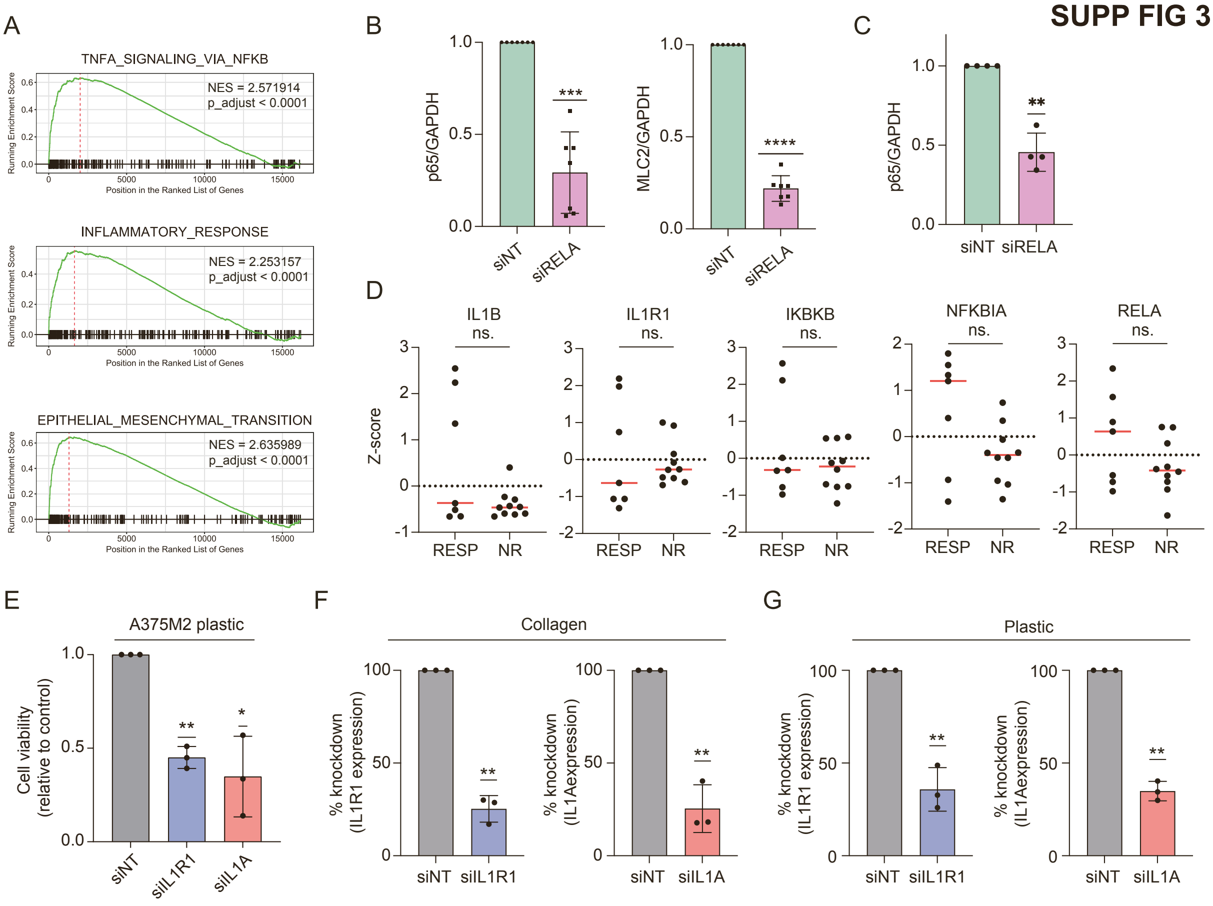

Fig. S3. NFKB activity is associated with ROCK inhibitor responses in non-epithelial solid tumors

(A) GSEA plots of selected processes from figure 3D.

(B) Quantification from figure 3I for p65/GAPDH and MLC2/GAPDH. Statistical test one sample t test. Bar plot shows mean value with whiskers showing SD. n=7 biological replicates.

(C) Quantification from figure 3J for p65/GAPDH. Bar plot shows mean value with whiskers showing SD. Statistical test one sample t test. n=4 biological replicates.

(D) Comparison of z-score expression between ROCK inhibitor responder and ROCK inhibitor non-responder melanoma cell lines. Statistical test unpaired t test. Plots show all points; red bar represents median value. n=7 cell lines (responders) and n=10 cell lines (non-responders).

(E) Quantification of A375M2 cell survival after two sequential rounds of transfection on plastic with siIL1A or siIL1R1. Bar plot shows mean value with whiskers showing SD. Statistical test: one sample t test to control. n=3 biological replicates.

(F) IL1R1 and IL1A mRNA levels measured by qPCR in siNT, siIL1R1 and siIL1A conditions. Bar plot shows mean value with whiskers showing SD. Statistical test one sample t test. n=3 biological replicates.

(G) IL1R1 and IL1A mRNA levels measured by qPCR in siNT, siIL1R1, siIL1A and siIL1A/IL1R1 conditions. Bar plot shows mean value with whiskers showing SD. Statistical test one sample t test. n=3 biological replicates.

For (B), (C), (D), (E), (F), (G): ns = not significant, * $p < 0.05$, ** $p < 0.01$, *** $p < 0.001$, **** $p < 0.0001$.

SUPP FIG 4

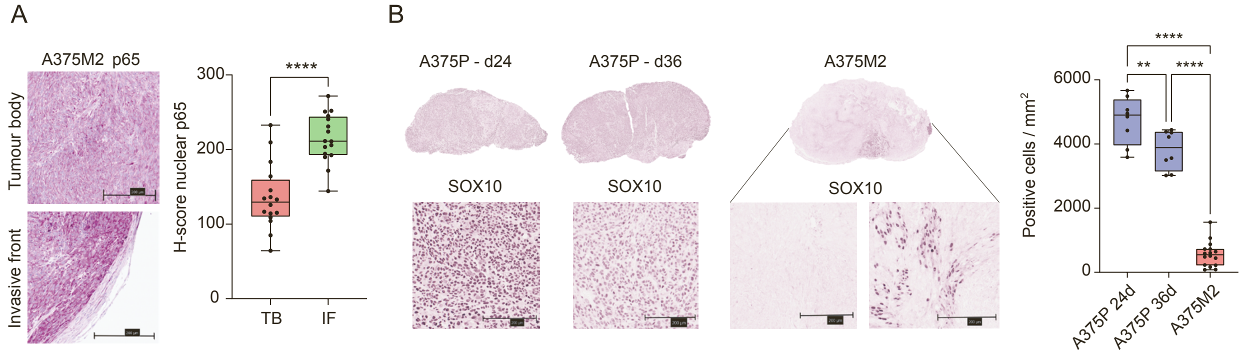

Fig. S4. ROCK and NFκB crosstalk *in vivo*

(A) Representative images of A375M2 stained for p65 from the tumor body and the invasive front (left) and quantification of the percentage of nuclear p65 (right). Box shows median value with interquartile range with whiskers showing min to max value; all points shown. Statistical test unpaired t test. n=16 tumors; TB and IF quantified within each tumor.

(B) Representative pictures from tumors derived from A375P at two different timepoints (24 and 36 days) and A375M2 after 24 days stained for SOX10 differentiation marker (left). Box shows median value interquartile range with whiskers showing min to max value; all points shown. Statistical test ordinary one-way ANOVA multiple comparisons comparing the mean of each column against the mean of every other column. Scale bar = 200µM. n=8 tumors (A375P d24), n=8 tumors (A375P d36) and n=16 tumors (A375M2).

For (A), (B): ** $p < 0.01$, *** $p < 0.001$, **** $p < 0.0001$.

SUPP FIG 5

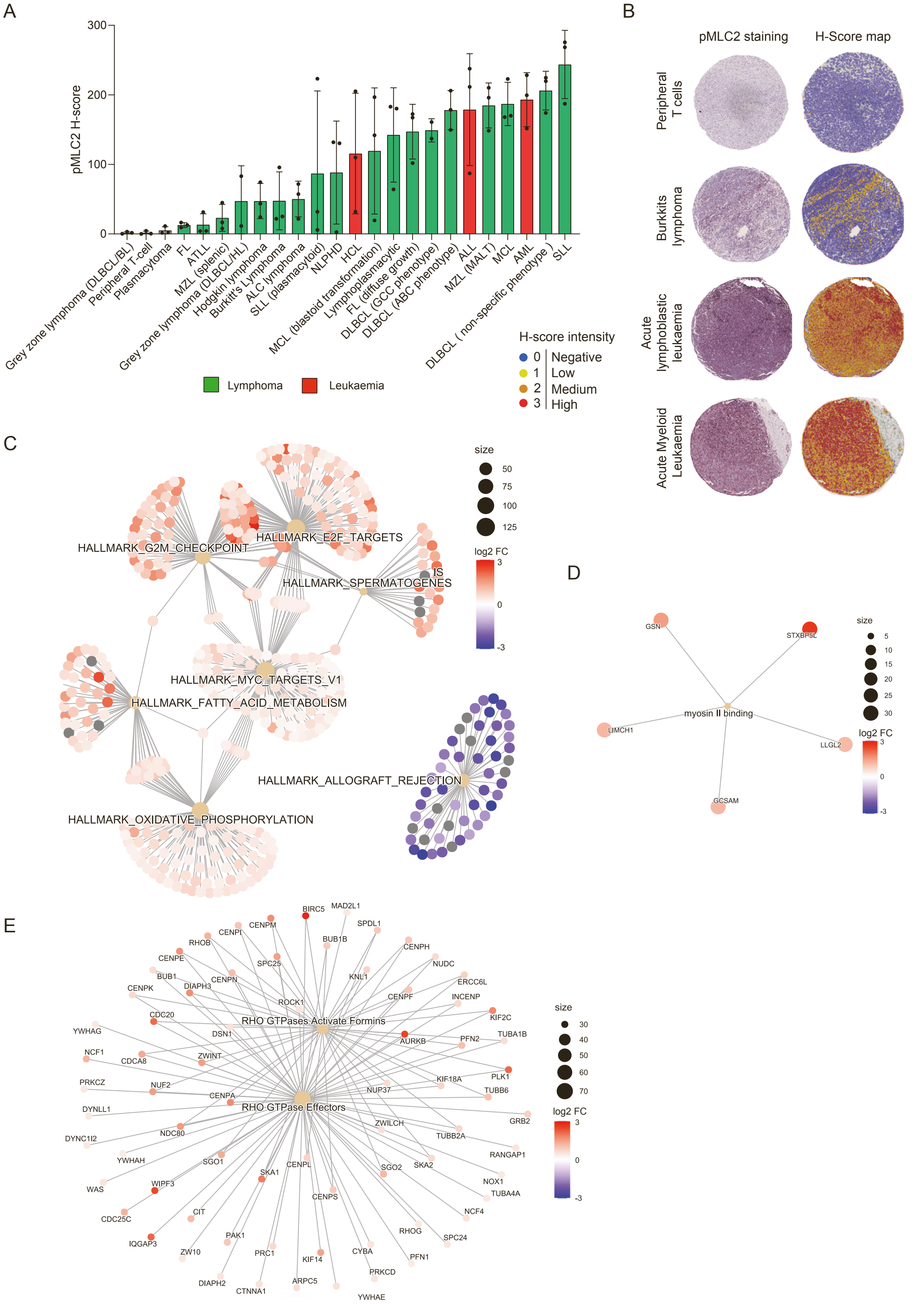

A
pMLC2 H-score
0
100
200
300
Grey zone lymphoma (DLBCL/BL)
Peripheral T-cell
Plasmacytoma
FL
ATLL
MZL (splenic)
Grey zone lymphoma (DLBCL/HL)
Hodgkin lymphoma
Burkitt's Lymphoma
ALC lymphoma
SLL (plasmacytoid)
NLPHD
HCL
MCL (blastoid transformation)
Lymphoplasmacytic
FL (diffuse growth)
DLBCL (GCC phenotype)
DLBCL (ABC phenotype)
ALL
MZL (MALT)
MCL
AML
DLBCL (non-specific phenotype)
SLL
Lymphoma
Leukaemia
B
pMLC2 staining
H-Score map
Peripheral T cells
Burkitts lymphoma
Acute lymphoblastic leukaemia
Acute Myeloid Leukaemia
H-score intensity
0 Negative
1 Low
2 Medium
3 High
C
HALLMARK_G2M_CHECKPOINT
HALLMARK_E2F_TARGETS
HALLMARK_SPERMATOGENESIS
HALLMARK_MYC_TARGETS_V1
HALLMARK_FATTY_ACID_METABOLISM
HALLMARK_ALLOGRAFT_REJECTION
HALLMARK_OXIDATIVE_PHOSPHORYLATION
size
50
75
100
125
log2 FC
3
0
-3
D
GSN
STXBP5L
myosin II binding
LIMCH1
LLGL2
GCSAM
size
5
10
15
20
25
30
log2 FC
3
0
-3
E
RHO GTPases Activate Formins
RHO GTPase Effectors
BIRC5
MAD2L1
SPDL1
CENPM
CENPI
BUB1B
RHOB
CENPH
CENPE
SPC25
NUDC
BUB1
DIAPH3
CENPN
KNL1
ERCC6L
CENPK
ROCK1
CENPF
INCENP
CDC20
KIF2C
YWHAG
DSN1
AURKB
PFN2
TUBA1B
NCF1
CDCA8
ZWINT
PLK1
KIF18A
PRKCZ
NUF2
NUP37
TUBB6
CENPA
DYNLL1
GRB2
NDC80
ZWILCH
DYNC1I2
TUBB2A
YWHAH
RANGAP1
SGO1
CENPL
SKA1
SGO2
SKA2
NOX1
WAS
WIPF3
TUBA4A
CENPS
CDC25C
CIT
NCF4
IQGAP3
RHOG
SPC24
PAK1
ZW10
PRC1
CYBA
KIF14
PFN1
PRKCD
DIAPH2
ARPC5
CTNNA1
YWHAE
size
30
40
50
60
70
log2 FC
3
0
-3

Fig. S5. Characterisation of AML response to ROCK inhibitors

(A) pMLC2 staining score from hematological malignancies tumor microarray (TMA). Box plots show median value with whiskers showing min to max. Green: lymphomas; red: leukaemias. Bar plot shows mean value with whiskers showing SD. n=2/3 human samples per tumour type.

(B) Representative images of pMLC2 staining and H-score analyzed in Qupath for peripheral T cells, Burkitt's lymphoma, ALL and AML.

(C) Gene-concept networks from GSEA comparing ROCK inhibitor responder vs ROCK inhibitor non-responder samples using the Hallmarks gene sets. Dots show genes contributing to the enrichment of the term and their associated log2 fold change. Categories shown represent top eight Hallmark terms by p-adjust.

(D) Gene-concept network of term myosin II binding from the Gene Ontology Molecular Function ontology.

(E) Gene-concept network of selected processes RHO GTPases Activate Formins and RHO GTPase Effectors from the Reactome database.

SUPP FIG 6

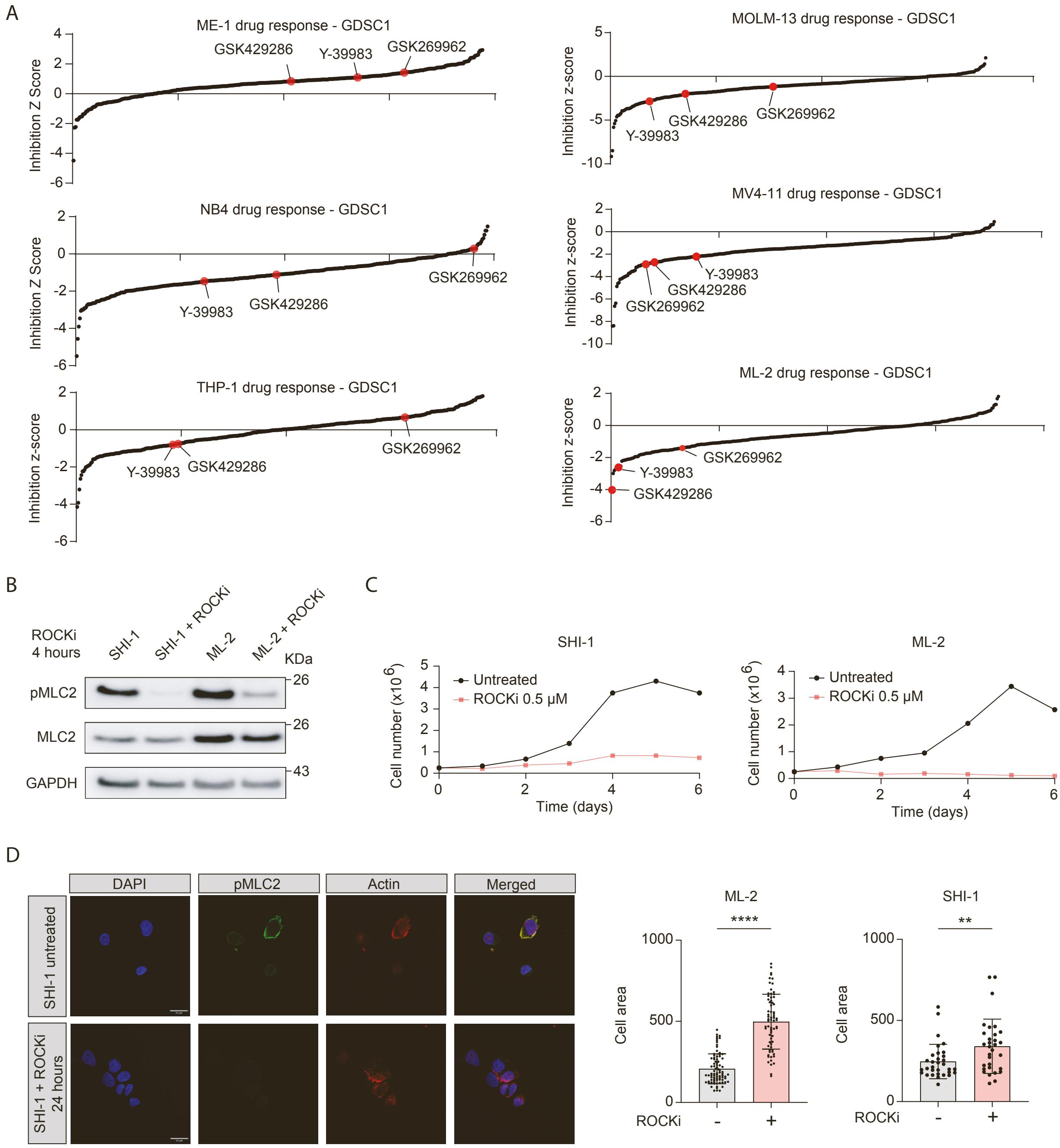

Fig. S6. ROCK inhibition has cytostatic and cytotoxic effects in AML cells

(A) Dot plot of inhibition z-score distribution of ML-2, MV4-11, MOLM-13, THP-1, ME-1 and NB4 from the GDSC2 screening of 358 inhibitors. Red dots indicate the three available ROCK inhibitors reported to exclusively target ROCK: GSK269962, GSK429286 and Y-39983. Each dot represents one inhibitor in the GDSC dataset.

(B) Western Blot for MLL-AF6 cell lines untreated or treated with 0.1 μM of ROCK inhibitor GSK269962 for four hours. Blotting for pMLC2, total MLC2 and GAPDH. n=3 biological replicates.

(C) Cell count of untreated or 0.5 μM ROCK inhibitor treated cells daily for six days.

(D) Immunofluorescence pictures of SHI-1 untreated or treated with 0.1 μM of ROCK inhibitor GSK269962 for 24 hours. Stained for DAPI (405), pMLC2 Ser19 (546) and Phalloidin/Actin (647) (left) and quantification of the levels of cell area for ML-2 and SHI-1 (right). Scatter dot plot shows mean value with whiskers showing SD. Statistical test: unpaired t test. For ML-2: n=83 cells (untreated) and n=70 cells (ROCKi). For SHI-1: n=32 cells (untreated) and n=31 cells (ROCKi). ** $p < 0.01$, **** $p < 0.0001$.

Data S1_Raw microscopy images

Figure 2

Figure 2G

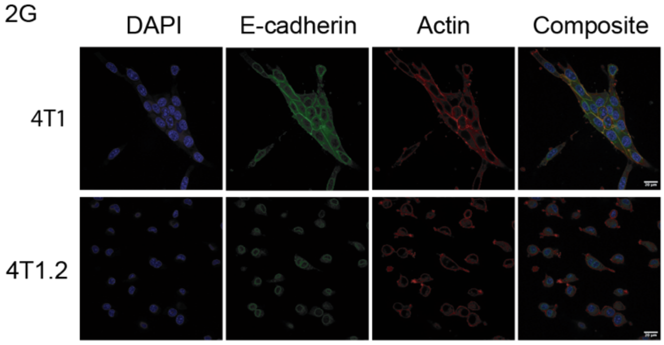

Figure 2J

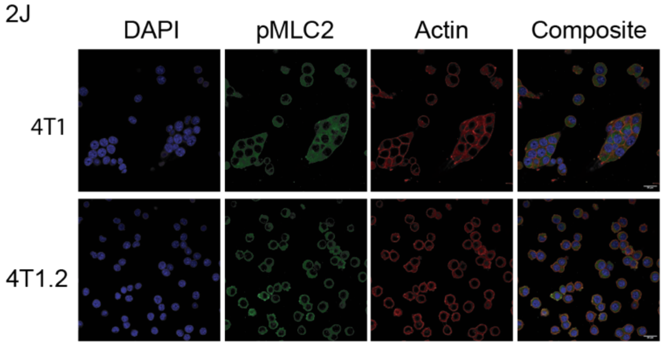

Figure 2M

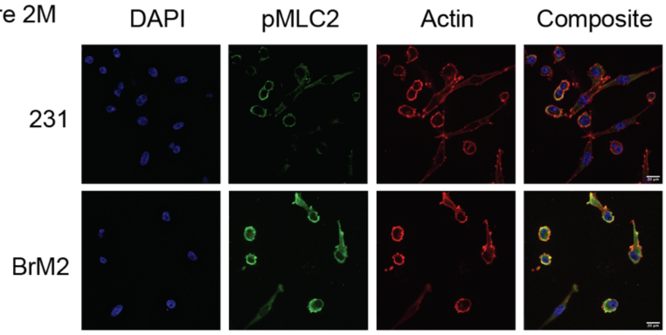

Figure 3

Figure 3A

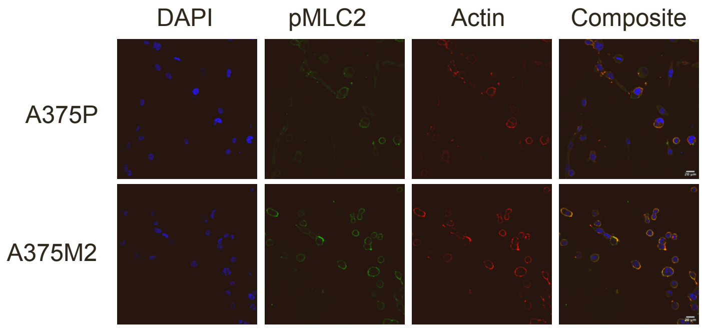

Figure 3F

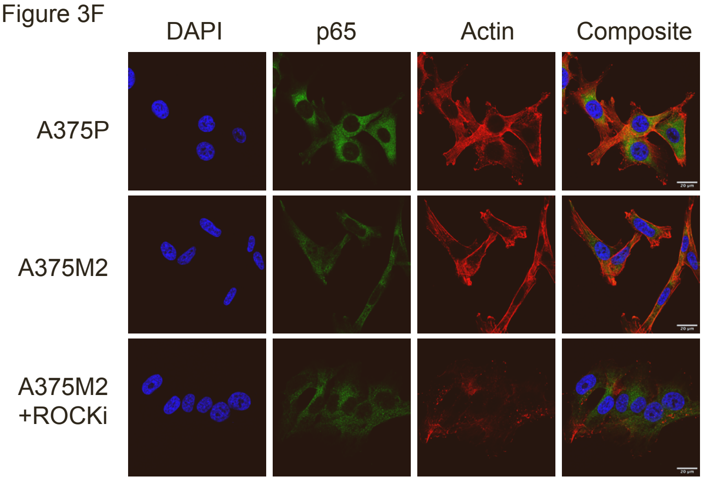

Figure 6 and supplementary figure 6

Figure 6C

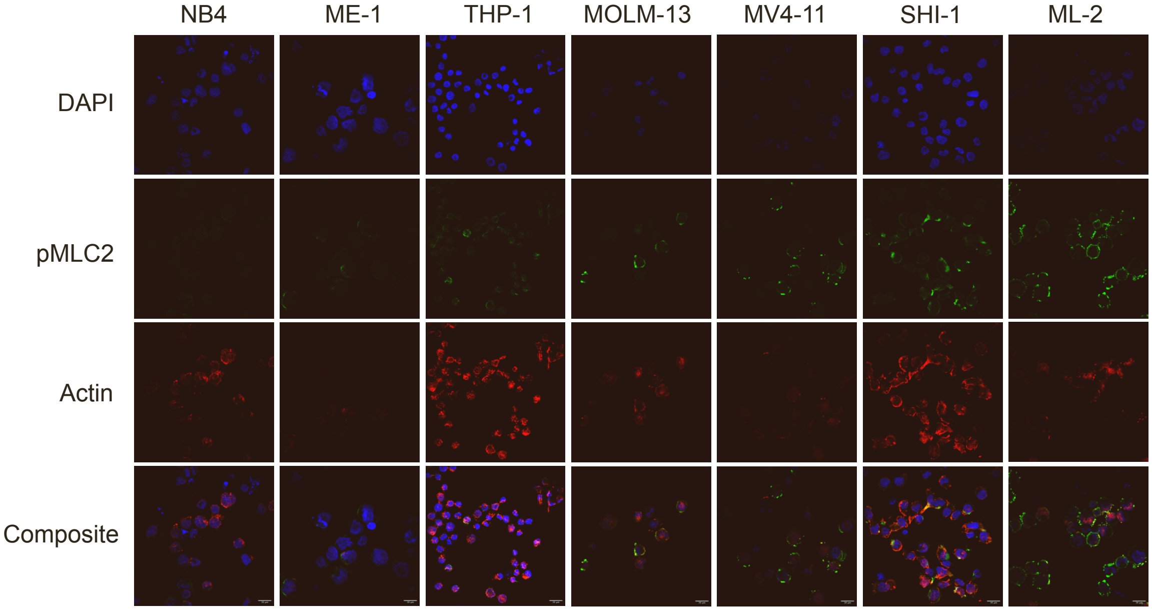

Figure 6D

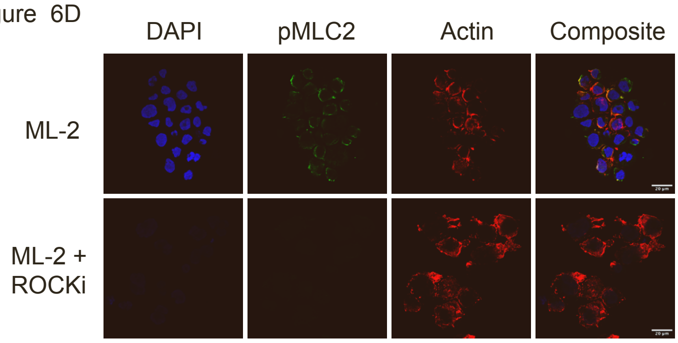

Supplementary Figure 6D

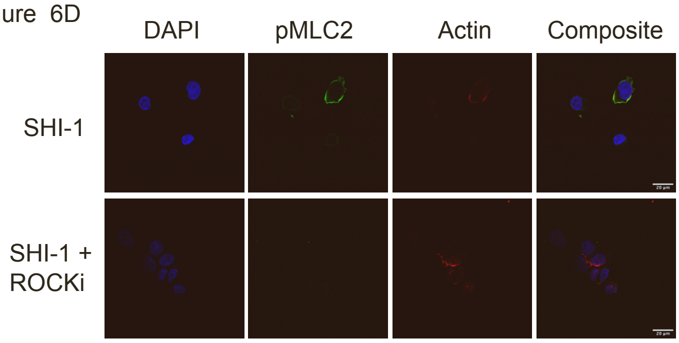

Supplement: Document S1. Figures S1–S6 and Data S1 [file mmc1.pdf]
